# From GWAS to function: lessons from blood cells

**DOI:** 10.1111/voxs.12217

**Published:** 2015-10-07

**Authors:** L. J. Vasquez, A. L. Mann, L. Chen, N. Soranzo

**Affiliations:** ^1^ Wellcome Trust Sanger InstituteWellcome Trust Genome CampusHinxtonUK; ^2^ Department of HaematologyUniversity of CambridgeCambridge Biomedical CampusCambridgeUK

**Keywords:** blood traits, function, genetic association, haematopoiesis

## Abstract

Haematopoiesis, or the process of formation of mature blood cells from committed progenitors, represents an accessible and well‐studied paradigm of cell differentiation and lineage specification. Genetic association studies provide a powerful approach to discover new genes, biological pathways and mechanisms underlying haematopoietic development. Here, we highlight recent findings of genomewide association studies (GWAS) linking 145 genomic loci to traits affecting the formation of red and white cells and platelets in European and other ancestries. We present strategies to address the main challenges in GWAS discoveries, particularly to find functional and regulatory effects of genetic variants, and to identify genes through which these genetic variants affect haematological phenotypes. We argue that studies of haematological trait variation provide an ideal paradigm for understanding the function of GWAS‐associated variants owing to the accessible nature of cells, simple cellular phenotype and focused efforts to characterize the genetic and epigenetic factors influencing the regulatory landscape in highly pure mature cell populations.

## Introduction

Haematopoiesis is the process whereby self‐renewing haematopoietic stem cells (HSC) in the bone marrow differentiate to lineage‐committed erythroid, myeloid and lymphoid progenitor cells 1. These progenitor cells will undergo successive differentiation steps to produce mature blood products such as thrombocytes (platelets), erythrocytes (red cells) and white cells. Blood is among the most accessible organs in the human body, from which pure individual cell populations can be isolated with relative ease compared to other human organs. In addition, the evolutionary conservation of hematopoietic processes facilitates the study of these mechanisms in model organisms 1.

Measurements of full blood counts (FBC), obtained through automated haematology analysers, including the size, physical characteristics or number of blood cells, have medical importance. Deviation from normal parameter ranges can be diagnostic for human diseases, indicating the presence of infection, anaemia, thrombotic diseases or haematological disorders 2, 3. Variation in blood cell traits has also been shown to be heritable, associated with genetic polymorphisms in human populations, and correlated to increased risk of certain diseases such as obesity, stroke and cardiovascular events such as coronary heart disease 4, 5, 6, 7, 8, 9, 10.

Genomewide association studies (GWAS) assess the statistical association of genetic variants with a given disease or trait of interest. GWAS in the last decade has successfully discovered thousands of genetic variants, mostly single nucleotide polymorphisms (SNPs), associated with the many common human diseases and traits 11. While this approach has been extremely fruitful in discovering novel loci, several challenges exist in the interpretation of GWAS findings. Studies have shown that a large proportion of identified SNPs map to non‐coding regions of the genome, where it is not straightforward to assign a functional mechanism to genetic variants 12. Furthermore, owing to linkage disequilibrium (LD), many genetic variants are typically associated with the phenotype at any given genomic locus, hindering efforts to identify the exact variant responsible for the effect (causal variant) 13.

Developing and implementing approaches to aid the interpretation of causal SNPs, and assigning a functional mechanism for how each variant alters a phenotype or disease state, represent an important present challenge to the field. Demonstrating potential functionality to trait‐associated variants is a necessary condition for definitive assignment of causality. Therefore, to reflect the difficulty in identifying causal variants, we refer to putative causal candidates as functional variants for the rest of this review.

Here, we highlight recent breakthroughs in understanding the genetic factors determining blood cell formation. We discuss strategies and challenges in prioritizing most likely affected genes and functional genetic variants. Finally, we discuss future opportunities in association studies involving blood traits.

## Findings from genomewide association studies of haematological traits

Here, we have surveyed the findings of 24 published GWAS studies in European (EUR) 3, 14, 15, 16, 17, 18, 19, 20, 21, 22, 23, 24, 25, Asian (ASN; Chinese, Japanese, Korean, South Asians) 26, 27, 28 and African (AFR) or African American (AA) 16, 29, 30, 31, 32 ancestries, isolate founder populations (Sardinia 33 and Iceland 34) and disease cohorts with sickle cell and beta‐thalassaemia anaemia 33, 35 (summarized in Table S1). Overall, there are approximately 145 genomic loci that are reported to be significantly associated with 15 different haematological indices (see Table 1). Most SNPs reported to date identify common genetic variants, defined as having minor allele frequency of 5% or above in the discovery population. They have been predominantly reported in populations of European ancestry (227 SNPs discovered, more than 62 000 study participants) compared to Asian (48 SNPs, 16 000 individuals) and African American (36 SNPs, 14 000 individuals) cohorts. Owing to the high correlation observed between different blood indices, GWAS variants are often reported as associated with multiple traits. Such variants may have an indirect effect, or act independently on each correlated trait (pleiotropy). Differentiating between direct and indirect effects will require the application of *ad hoc* statistical approaches for instance multivariate modelling 36.

**Table 1:**
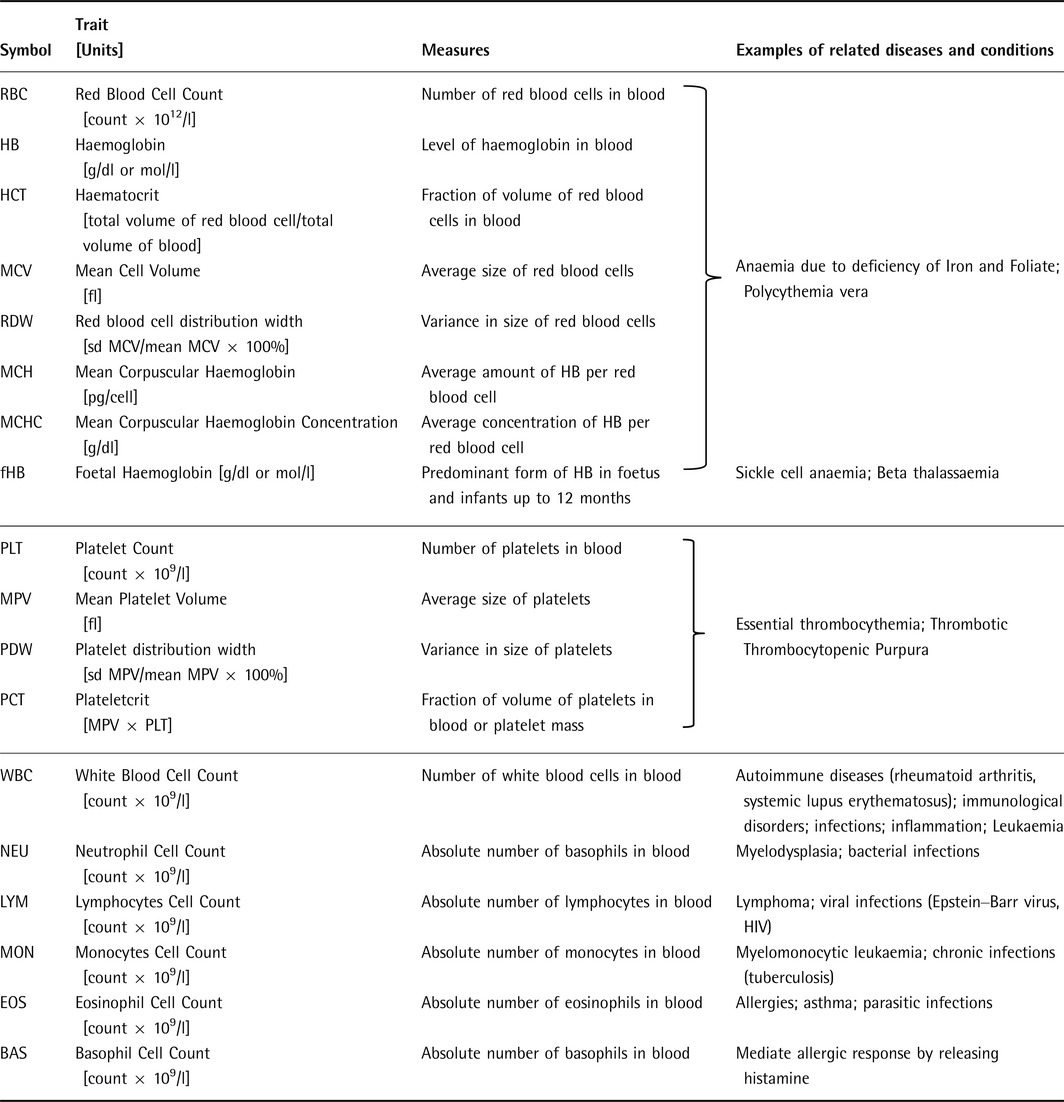
Summary of the main haematological indices, unit of measure and related diseases and conditions

As for other complex traits, we found that GWAS findings for haematological indices predominantly map to non‐coding regions of the genome (Table S1). Genes closest to the association peaks were enriched for genes regulating haematological functions 14, 15, and for genes causative for Mendelian blood disorders (Tables S1–S2) such as haemolytic anaemia (*HK1, G6PD*), sickle cell disease (*BCL11A, HBB, HBSx1L‐MYB*), thrombocytopenia (*MPL*), leukaemia (*PTPN11*) and bone marrow failure (*TERT*). Furthermore, genes in nearby regions are enriched for relevant Gene Ontology biological processes such as haematopoiesis (FDR ≤ 1E‐3; genes involved in the process are *RUNX1, TAL1*), immune system development (2E‐3; *IFl16*,* PTPRC*) and oxygen transport (8E‐2; *HBQ1, HBA1*). Follow‐up of early genetic association studies has revealed novel regulators of haematopoiesis 14, 15, 33, 37. For instance, the largest GWAS to date in red cells and platelets 14, 15 have led to the discovery of 66 novel genes with validated haematopoietic phenotypes in model organisms.

Despite successful gene discoveries, blood GWAS only explain a fraction (4–10%) of baseline differences of measured blood traits in the population 14, 15. In addition, study of parameters for myeloid and lymphoid white blood cell subtypes encompassing important functions in host defence, immunity and inflammation has been hampered by a lack of suitable data in highly powered cohorts (Table S1 for existing studies). Hence, the challenge now is to increase sample size, sequencing resolution and number of measured traits so as to discover more associations. Current discovery efforts based on large‐scale cohorts (e.g. UK Biobank 38 and INTERVAL study 39) or collaborative efforts based on bespoke genotyping arrays 40 should increase the power of discoveries, alongside whole‐genome sequencing efforts (e.g. UK10K project).

## Strategies for selecting candidate genes associated with GWAS

**Figure 1:**
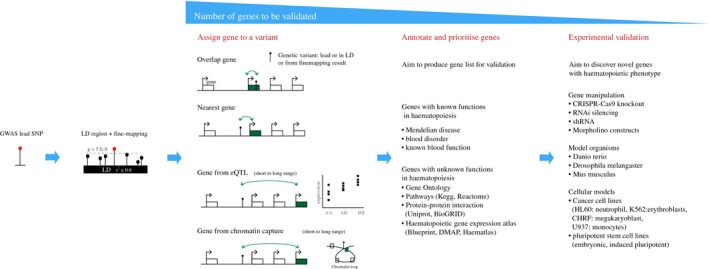
Strategies employed to prioritize gene targets. Summary of the main approaches that can be used for assigning genes to a genetic variant identified from GWAS and fine mapping approaches, and experimental approaches that can be used to validate the hypothesis that given gene candidates are influenced by the variant of interest. For references relating to techniques please see Table S3.

To realise the translational benefit of GWAS studies, it is essential to identify the target genes through which identified variants influence traits or phenotypes. This can lead to the discovery of new genes and pathways involved in biological processes or identify those that underlie risk to particular diseases. Here, we outline general strategies in assigning gene targets to GWAS variants and in prioritizing genes for experimental validation (Fig. 1), highlighting where they have been successfully applied to blood cell studies.

We discussed in the previous section how genes proximal to GWAS variants may be prime candidates for further investigation, particularly those with a known function relating to the trait of interest. However, a potential mechanism through which non‐coding SNPs are believed to act is through the disruption of regulatory elements influencing distally located genes. This implies that the nearest gene is not always the target gene mediating the genetic association 41, 42. For instance, regulatory enhancers can also interact with promoters of distal genes and can ‘skip’ over nearest genes to regulate those situated at further distances or in *trans* 43. In this context, we discuss two methods that probe short‐ and long‐range interactions between a variant and the target gene.

Expression quantitative trait loci (eQTL) mapping is performed to find statistical association between a genetic variant and the transcript level of a gene considered as a quantitative trait 44. eQTL studies can be used as a general method to help identify a set of target genes as many SNPs associated with GWAS traits were shown to be eQTLs 15, 45. As an example, GWAS SNP rs342293 (associated with platelet volume) was found to influence the mRNA levels of *PIK3CG* kinase gene in platelets and macrophages 24, 46. This SNP is located in a megakaryocyte‐specific open chromatin region 46 and causes differential binding of the transcription factor EVI1. Still, there are inherent limitations to assigning genes through eQTL studies. Even though most eQTL SNPs are proximal to transcription start sites (TSS) of their target genes 47, more complex cis‐ and trans‐ effects with co‐regulation of multiple genes are relatively common. Analyses of promoter and chromatin interactions in relevant tissues can be used to provide additional evidence to assign target genes to each QTL. Secondly, the statistical cost of multiple testing implies that most current studies have limited statistical power to detect effects in *trans*.

Thus, more accurate methods of target gene identification are required. Recent advances in chromatin conformation techniques provide such opportunities. Chromatin conformation capture (3C) and variants of this approach (4C, 5C, Hi‐C and ChIA‐PET) probe long‐range interactions by utilizing formaldehyde‐directed cross‐linking of genomic modules that are close in physical space 48. For example, using ChIA‐PET and 5C, GWAS variants located in open chromatin (DNAse‐I hypersensitive sites) were found to control distant genes associated with relevant phenotypes 12. Specifically, the SNP rs385893 associated with platelet count is located in a DHS site and physically interacts with its target gene, *JAK2*, which plays an important role in platelet formation with mutations in this gene being associated with myeloproliferative disorders 12. Further development in this area now enables the high‐throughput, genomewide application of these techniques to assigning gene targets to variants. Novel methods such as Capture‐C and Capture‐HiC enable simultaneous assessment of genomewide SNP targets through the addition of an enrichment step using probes to select defined regions (known often as ‘baits’) 49, 50. Capture‐HiC has been applied to assay the interactions of the genomewide cellular complement of promoters 50. Like eQTLs, chromatin interactions are context‐dependent, and thus, the cellular background in which these interactions are probed needs to be considered.

With a list of candidate target genes for each GWAS SNP, it is useful to annotate and then prioritize genes for experimental validation using approaches summarized in Fig. 1. To validate whether a gene causes the phenotype of interest, genetic manipulation techniques such as CRISPR/Cas9 and gene knockdown approaches in model organisms and/or cellular models may be applied 51, 52 (Fig. 1). A recent GWAS study has demonstrated platelet phenotype of 11 novel genes by silencing them in model organisms 14. Antisense morpholino oligonucleotide‐directed silencing of one such gene, the *ARHGEF3* ortholog in zebrafish (*Danio rerio*), leads to ablation of both primitive erythropoiesis and thrombocyte formation, and a novel role in the regulation of iron uptake and erythroid cell maturation 14, 53. In‐depth modelling of haematopoietic phenotypes can also be achieved in model organisms. For instance, using *in vivo* imaging of the transparent zebrafish embryo, the developmental stages of haematopoiesis are easily traceable from primitive to adult haematopoiesis 37.

## Strategies for selecting candidate variants associated with GWAS

We have discussed methods for prioritizing gene targets where genes are either mapped to the lead SNP or to any variants within an LD region. However, the lead SNP is not necessarily the functional variant. Therefore, without appreciating this, it is possible that genes will be mapped to variants that may not be causally responsible for the phenotypic change. In addition, phenotypic differences could also be driven by a combination of variants. It is therefore important to identify which variants are functional to explain the molecular mechanisms underlying genetic associations.

**Figure 2:**
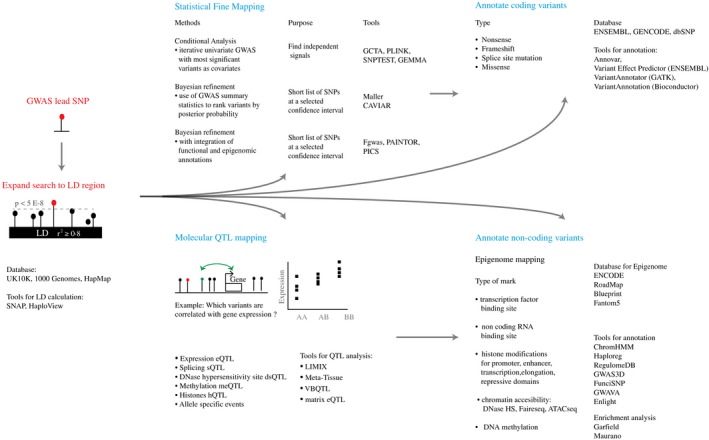
Strategies employed to prioritize functional variants. Trait‐associated variants and variants in high LD can be further defined through statistical fine mapping approaches. Methods to annotate variants can vary depending on the location of the variant (non‐coding versus coding). Demonstrating potential functionality through functional approaches is necessary to infer variant causality and the mechanism underlying the association. For references relating to techniques please see Table S3.

Extensive linkage disequilibrium in the human genome and the incomplete ascertainment of sequence variation in genotyping arrays make it difficult to distinguish between independent genetic contributions. We outline in Fig. 2 the strategies in prioritizing variants that are likely to underlie causality by identifying regulatory effects or functionality associated with specific variant candidates. From the GWAS lead SNP, the search is expanded to take all variants in high LD (e.g. r^2^ ≥ 0·8), that is variants that are highly correlated with the lead SNP. For this purpose, it is recommended to use the haplotype reference of the discovery population. A first intuitive step is to assess whether a variant overlaps a coding region, which potentially leads to amino acid sequence alterations. Changes to protein sequence can in turn influence phenotype, thus indicating that a variant may be functional. However, an altered protein is not always causative and a change in amino acid sequence may not always change protein function.

To further refine association signals within the LD region, we briefly describe in Fig. 2 the statistical methods used in fine mapping genetic variants. These approaches can significantly eliminate proxy effects and reduce the list to the most probable groups of trait‐associated variants with independent effects. There are however limitations to these methods. Conditional regression may miss identifying functional variants when variants are in perfect LD, whereas Bayesian methods 54 may only assume a single functional variant in a locus. Nevertheless, Bayesian scoring can incorporate genomic annotations (e.g. transcription start sites) and epigenetic data (e.g. enhancer histone modifications) to set prior weights in ranking variants 55, 56, 57.

Assigning functional characteristics to variants within a LD region can help to indicate causality. However, the non‐coding location of a high proportion of reported complex trait GWAS SNPs complicates assignment of molecular mechanism due to our incomplete understanding of the function of large regions on the non‐coding genome. This is where epigenetic information and knowledge of the function of genomic architecture can be valuable in the generic annotation of non‐coding variants, notably those that are in gene deserts. In addition, a variant could be located hundreds of kilobases away in linear scale from the target gene but due to chromatin looping, it is spatially close to directly regulate gene expression. Epigenetic markers such as histone modifications can mark transcriptional activity (H3K4me3, H3K36me3), cis and distal enhancer regions (H3K4me1, H3K27Ac), and repressed genes (H3K27me3, H3K9me2/3). Genomic assays such as DNAse‐seq and ATAC‐seq can identify open chromatins that may be bound by transcription factors or repressors (e.g. CTCF). Sequence variation at the nucleotide level can add or remove the methylation of a nucleotide and for instance can disrupt binding of transcription factors (e.g. CTCF) especially if found in CpG islands 58. There are already available tools (Fig. 2) that can easily integrate GWAS variants with large‐scale epigenome data (e.g. ENCODE) and in addition to providing LD information can also rank variants according to cumulative evidence of regulatory marks. Lastly, analysis can also be focused on individual variants and their association to epigenetic marks as molecular traits within the context of quantitative trait loci (QTL) study and allele‐specific analysis (Fig. 2) 59. Recently, alternative splicing has been shown to influence transcriptional diversity in haematopoietic progenitors in a cell specific manner 60. Therefore, new efforts in treating splicing as a quantitative trait (sQTL) may reveal novel loci. QTL mapping can provide direct evidence of *cis* and distal regulation of sequence variation affecting differences in epigenetic regulation, with the aim to link to transcriptional and phenotypic effect.

Epigenetic and regulatory information does, however, differ based on cell‐type and developmental or other context, so availability of epigenetic data for cell types that are most relevant to the phenotype or disease of interest can greatly enhance the interpretation of functional consequences of GWAS variants 12, 55, 61. Enrichment analysis (Fig. 2) is designed to rank and evaluate which combinations of tissue/cell and functional annotation types are most informative for a given phenotype of interest 12, 55, 61. There are numerous studies 1, 62 using immortalized cancer cell lines (e.g. LCL, CHRF, HL60) as model blood cells. However, the epigenome of such cells has been demonstrated to be different from primary cells, for instance altered DNA methylation in LCLs 63, 64. More recently, the BLUEPRINT Project 65 has been generating reference epigenome data for primary blood cell types isolated from healthy blood donors and for selected disease population. Future efforts in this field will provide insights into how cellular specificity, developmental stage or response to external stimuli all impact these quantitative traits.

Integration with these annotated regulatory genomic features will be important to suggest hypotheses by which potential functional variants may impact phenotype/traits through regulatory effects, but these must be subsequently experimentally tested. As an example, variant rs2038479 in LD with MPV associated lead SNP rs10914144 was validated to mark an alternative promoter site affecting transcription of gene *DNM3* and consequently leading to reduced proplatelet formation *in vitro* 66. The variant rs2038479 was prioritized for a functional follow‐up experiment because it was found in a MK‐specific open chromatin region that co‐localizes binding of megakaryocytic transcription factor MEIS1, altogether a genomic evidence which suggests the mechanism of how this variant regulates platelet phenotype.

Variants are often described as enriched with enhancer or promoter marks and *in vitro* cellular assays can directly demonstrate whether a variant possesses enhancer or promoter activity through using luciferase reporter systems 67. Transgenic mouse assays enable an *in vivo* assessment of enhancer activity 68. Massively parallel reporter assays extend this approach to assay thousands of variants for enhancer activity 69. Recent, larger scale, higher throughput assays of enhancer activity include techniques FIREWACh 70 and STARR‐seq 71. STARR‐seq, applied to the *Drosophila* genome, uses RNA‐seq based readouts to measure enhancer strength and genomic location. Alternatively, FIREWACh qualitatively assays nucleosome‐free regions of the mammalian genome. In future, it may be possible to adapt these techniques in order to experimentally estimate the proportion of non‐coding variants that possess enhancer activity, thus suggesting potential mechanisms in high‐throughput experiments.

Interaction of a variant sequence with a protein can be indicative of function, and disruption of these binding sites can influence gene expression. *In vitro* gel shift experiments can demonstrate interaction with specific proteins 67. Genomewide *in vivo* TF binding is assayed using ChIP‐seq, if cells from the individual with the desired genotype are available through recall‐by‐genotype or the generation of iPSC lines 67. Alternatively, within one (heterozygous) individual, allele‐specific approaches can be used to investigate variant functionality 64, 72.

## Current challenges and future opportunities in Blood GWAS

### Rare, low frequency and copy number variants from whole‐genome sequencing

GWAS studies in complex traits and diseases including blood phenotypes and disorders have until now mainly targeted genetic variants that are relatively common in the general European population (MAF>5%). However, associated common variants across all traits have only accounted for less than 10% of genetic heritability in blood cell traits, despite large sample sizes and dense genotyping. With decreases in cost of whole‐genome and whole‐exome sequencing, the reach of association studies should soon extend to low frequency and rare variants with intermediate to large effect sizes, and more exhaustive evaluations of structural variation (e.g. insertions, deletions, duplications) 73. Whole‐genome sequencing will allow association tests for variants across the full allelic spectrum and is also expected to greatly increase the resolution of imputation‐based analysis through the generation of enhanced imputation panels. This initiative is being exemplified by large‐scale genetic studies such as the UK10K project and in addition studies with much larger sample cohorts and extensive meta data such as UK Biobank 38. For instance, the UK Biobank as a major national health bioresource aims to genotype data for 500 000 volunteers and to record extensive haematological measures and lifestyle information. While these large‐scale initiatives are expected to greatly increase the pace of genetic discoveries in the near future, it is yet unclear what prospects there are for clinically translating GWAS findings, as the vast majority of variants have neither well‐defined biological nor clinical implications despite the widespread use of blood indices as biomarkers for diseases.

### Current efforts in the epigenome of human blood

New studies suggest that epigenetics and not genetics may contribute a substantial component of trait heritability 74, 75. Whether this is true or not, addressing the lack of data in the epigenome of human primary cells including blood tissues has been the motivation of various consortia such as NIH Roadmap 76 and Blueprint 65. We now know that epigenetic data are necessary to elucidate cell specific regulatory mechanisms that control phenotypes 77 and severity of diseases and could suggest new drug targets for therapeutic disease treatments 65. Recently, the NIH Roadmap released the largest catalogue of 111 human reference epigenomes in at least 24 different tissues, including 8 blood cell types 76. Ongoing efforts in the Blueprint consortium aim to provide the first extensive reference epigenome (up to 100) of the human haematopoietic tree covering more than 50 high‐quality purified distinct primary blood subtypes from healthy individuals and their malignant leukaemic counterparts 65. However, there is still a lack of sufficient data that interrogate the direct chromatin interaction between putative enhancers and their target gene promoters to finally validate long‐range gene regulation. There is a need therefore of corresponding high‐quality genomewide chromatin capture data such as Hi‐C.

### Pluripotent stem cell‐derived blood as a model system of haematopoiesis

Advancing technologies to expand and differentiate pluripotent stem cells into various somatic tissues including blood cells (e.g. megakaryocytes/platelets 78, erythroid progenitors/RBC 79, macrophages 80) for clinical and commercial applications opens unprecedented opportunities to capitalize on the availability of these novel cells as a model system of haematopoiesis. There is potential to produce and bank all blood subtypes especially those rare populations, including genome‐edited mutations. The effect of variation can then be studied from the start of differentiation with HSCs towards production of mature blood cells. Although the differentiation protocol, which is still a work in progress, tries to recapitulate *in vivo* HSC differentiation *in vitro*, the derived cells are not the exact equivalent of bone marrow‐derived blood cells in terms of their full genomic and epigenetic character and even functionality.

The genetic techniques we have described have identified many new regulators in processes such as haematopoiesis. With recent efforts from studies such as the INTERVAL study and UK Biobank, association studies of blood cell traits in very large cohorts (in the tens to hundreds of thousands) will provide the means to vastly increase the number of discovered loci. Full description of traits including white blood cell differentials also increases the power of these studies to discover new loci. We now have all the tools in place to improve our understanding of not only the haematopoietic system but also, more generally, the functional consequences of sequence variation and their contribution to complex human traits.

## Supporting information


**Table S1.** Summary findings of 23 published GWAS studies in haematological traits. For variant annotation, we used ANNOVAR and GENCODE.
