# Supplementary material for: From GWAS to function: lessons from blood cells
Source: ISBT Sci Ser. 2015 Oct 7;11(Suppl Suppl 1):211–9. doi: 10.1111/voxs.12217 (PMC4916502; doi:10.1111/voxs.12217)
Supplement: Supplementary file 1 — Table S1. Summary findings of 23 published GWAS studies in haematological traits. For variant annotation, we used ANNOVAR and GENCODE. [file VOXS-11-211-s001.docx]

**Supplementary Tables**

**Table S1.**  Summary findings of 23 published GWAS studies in haematological traits. For variant annotation, we used ANNOVAR and GENCODE.

| **Trait** | **Region** | **rsid** | **Chr** | **Position** | **Variant annotation** | **Nearest genes**  **(GENCODE)** | **PUBMED_ID** | **1000 Genomes Project Population** | **Discovery sample** | **OMIM trait and disease (Haematological related conditions are in green)** |
| --- | --- | --- | --- | --- | --- | --- | --- | --- | --- | --- |
| MCHC | 1p36.22 | rs1175550 | 1 | 3691528 | ncRNA_intronic | *RP1-286D6.2* | 23222517 | EUR | 62,553 European ancestry individuals, 9,308 South Asian ancestry individuals |  |
| MPV | 1p36.22 | rs17396340 | 1 | 10286176 | intronic | *KIF1B* | 22139419 | EUR | 18,600 European ancestry individuals | Charcot-Marie-Tooth disease; Pheochromocytoma; Neuroblastoma, susceptibility to |
| PLT | 1p36.22 | rs2336384 | 1 | 12046063 | intronic | *MFN2* | 22139419 | EUR | 48,666 individuals of European ancestry | Charcot-Marie-Tooth disease; Hereditary motor and sensory neuropathy VI |
| MCH | 1p34.3 | rs3916164 | 1 | 40069939 | intergenic | *PABPC4 (dist=27499), HEYL (dist=19886)* | 23222517 | EUR | 62,553 European ancestry individuals, 9,308 South Asian ancestry individuals |  |
| PLT | 1p34.2 | rs17292650 | 1 | 43803807 | nonsynonymous SNV | *MPL* | 23103231 | AFR | 767 African Americans | Thrombocytopenia, congenital amegakaryocytic; Thrombocythemia ; Myelofibrosis with myeloid metaplasia, somatic |
| MCV | 1p32 | rs741959 | 1 | 47676233 | intergenic | *PDZK1IP1 (dist=19517), TAL1 (dist=5730)* | 23222517 | EUR | 62,553 European ancestry individuals, 9,308 South Asian ancestry individuals | Leukemia-1, T-cell acute lymphocytic |
| MCHC | 1q22 | rs6684514 | 1 | 156255456 | nonsynonymous SNV | *TMEM79* | 20139978 | ASN | 14,377 Japanese ancestry individuals |  |
| MCHC | 1q23.1 | rs857684 | 1 | 158575729 | upstream | *OR10Z1* | 23222517 | EUR | 62,553 European ancestry individuals, 9,308 South Asian ancestry individuals |  |
| WBC | 1q23.1 | rs4657616 | 1 | 158971086 | intronic | *IFI16* | 23263863 | AFR | 7,943 African American children, 6,234 European ancestry children |  |
| WBC | 1q23.2 | rs2814778 | 1 | 159174683 | UTR5 | *DARC* | 22037903 | AFR | 62 American Indian or Alaska Native ancestry individuals, 158 Asian ancestry individuals, 3,272 African American ancestry individuals, 114 other ancestry individuals, 23,244 European ancestry individuals, 996 unknown ancestry individuals | Blood group, Duffy system; Malaria, vivax, protection against; White blood cell count QTL |
| WBC | 1q23.2 | rs12075 | 1 | 159175354 | nonsynonymous SNV | *DARC* | 22037903 | AFR | 62 American Indian or Alaska Native ancestry individuals, 158 Asian ancestry individuals, 3,272 African American ancestry individuals, 114 other ancestry individuals, 23,244 European ancestry individuals, 996 unknown ancestry individuals | Blood group, Duffy system; Malaria, vivax, protection against; White blood cell count QTL |
| WBC | 1q23.3 | rs2340727 | 1 | 161946727 | intergenic | *ATF6 (dist=12867), OLFML2B (dist=6255)* | 23263863 | EUR | 7,943 African American children, 6,234 European ancestry children |  |
| PLT | 1q24.3 | rs10914144 | 1 | 171949750 | intronic | *DNM3* | 22139419 | EUR | 48,666 individuals of European ancestry |  |
| MPV | 1q24.3 | rs10914144 | 1 | 171949750 | intronic | *DNM3* | 22139419/19820697 | EUR | 18,600 European ancestry individuals/4,627 European individuals |  |
| MCH | 1q31.3 | rs12127588 | 1 | 198595506 | intergenic | *RP11-553K8.2 (dist=27508), PTPRC (dist=12295)* | 20139978 | ASN | Up to 14,362 Japanese ancestry individuals | Hepatitic C virus, susceptibility to; Severe combined immunodeficiency, T cell-negative, B-cell/natural killer-cell positive |
| RBC | 1q32.1 | rs7529925 | 1 | 199007208 | ncRNA_intronic | *RP11-16L9.4* | 23222517 | EUR | 62,553 European ancestry individuals, 9,308 South Asian ancestry individuals |  |
| MCHC | 1q32.1 | rs7551442 | 1 | 203655121 | intronic | *ATP2B4* | 23222517 | EUR | 62,553 European ancestry individuals, 9,308 South Asian ancestry individuals |  |
| MPV | 1q32.1 | rs1668873 | 1 | 205235990 | intronic | *TMCC2* | 19820697 | EUR | 4,627 European individuals |  |
| PLT | 1q32.1 | rs1668871 | 1 | 205237137 | intronic | *TMCC2* | 22139419 | EUR | 48,666 individuals of European ancestry |  |
| MPV | 1q32.1 | rs1172130 | 1 | 205244953 | intergenic | *TMCC2 (dist=2482), NUAK2 (dist=26234)* | 22139419 | EUR | 18,600 European ancestry individuals |  |
| MCH | 1q32.1 | rs9660992 | 1 | 205249450 | intergenic | *TMCC2 (dist=6979), NUAK2 (dist=21737)* | 23222517 | EUR | 62,553 European ancestry individuals, 9,308 South Asian ancestry individuals |  |
| BAS | 1q32.1 | rs12748961 | 1 | 205676263 | intergenic | *SLC45A3 (dist=26676), NUCKS1 (dist=5684)* | 21738478 | ASN | 8,794 Japanese ancestry individuals |  |
| PLT | 1q44 | rs7550918 | 1 | 247675559 | intronic | *C1orf150* | 22139419 | EUR | 48,666 individuals of European ancestry |  |
| PLT | 1q44 | rs3811444 | 1 | 248039451 | nonsynonymous SNV | *TRIM58* | 22139419 | EUR | 48,666 individuals of European ancestry |  |
| RBC | 1q44 | rs3811444 | 1 | 248039451 | nonsynonymous SNV | *TRIM58* | 23222517 | EUR | 62,553 European ancestry individuals, 9,308 South Asian ancestry individuals |  |
| MCV | 1q44 | rs11204538 | 1 | 248046272 | intronic | *OR2W3* | 20139978 | ASN | 14,364 Japanese ancestry individuals |  |
| WBC | 2p24.1 | rs7600502 | 2 | 22297003 | ncRNA_intronic | *AC068490.2* | 22788528 | ASN | 8,722Korean |  |
| PLT | 2p23.3 | rs1260326 | 2 | 27730940 | nonsynonymous SNV | *GCKR* | 22139419 | EUR | 48,666 individuals of European ancestry | Fasting plasma glucose level QTL |
| MPV | 2p23.1 | rs649729 | 2 | 31464385 | intronic | *EHD3* | 22139419 | EUR | 18,600 European ancestry individuals |  |
| MPV | 2p23.1 | rs647316 | 2 | 31464829 | intronic | *EHD3* | 19820697 | EUR | 4,627 European individuals |  |
| PLT | 2p23.1 | rs625132 | 2 | 31482300 | intronic | *EHD3* | 22139419 | EUR | 48,666 individuals of European ancestry |  |
| PLT | 2p21 | rs17030845 | 2 | 43687879 | intronic | *THADA* | 22139419 | EUR | 48,666 individuals of European ancestry |  |
| RBC | 2p21 | rs10495928 | 2 | 46353166 | intronic | *PRKCE* | 20139978 | ASN | 14,392 Japanese ancestry individuals |  |
| HB | 2p21 | rs10495928 | 2 | 46353166 | intronic | *PRKCE* | 19862010 | EUR | 24,167 European ancestry individuals |  |
| HCT | 2p21 | rs4953318 | 2 | 46355051 | intronic | *PRKCE* | 23222517 | EUR | 62,553 European ancestry individuals, 9,308 South Asian ancestry individuals |  |
| HCT | 2p21 | rs13008603 | 2 | 46355848 | intronic | *PRKCE* | 23446634 | AFR | Up to 16,485 African American individuals |  |
| HCT | 2p21 | rs10168349 | 2 | 46360907 | intronic | *PRKCE* | 19862010 | EUR | 24,167 European ancestry individuals |  |
| MCV | 2p16.1 | rs2540917 | 2 | 60608759 | ncRNA_intronic | *AC007381.2* | 19862010 | EUR | 24,167 European ancestry individuals |  |
| MCV | 2p16.1 | rs243070 | 2 | 60620286 | intergenic | *AC007381.2 (dist=1776), BCL11A (dist=58016)* | 23222517 | EUR | 62,553 European ancestry individuals, 9,308 South Asian ancestry individuals |  |
| fHB | 2p16.1 | rs766432 | 2 | 60719970 | intronic | *BCL11A* | 20018918 | AFR | 848 Black cases |  |
| fHB | 2p16.1 | rs11886868 | 2 | 60720246 | intronic | *BCL11A* | 18245381 | EUR | 4,305Sardinians |  |
| EOS | 2q12.1 | rs1420101 | 2 | 102957716 | synonymous SNV | *IL1RL1* | 19198610 | EUR | 9,392 Icelanders |  |
| MCV | 2q13 | rs10207392 | 2 | 111849659 | intronic | *ACOXL* | 23222517 | EUR | 62,553 European ancestry individuals, 9,308 South Asian ancestry individuals |  |
| WBC | 2q21.3 | rs35940156 | 2 | 136575300 | nonsynonymous SNV | *LCT* | 23103231 | AFR | 766 AfricanAmericans | Lactase deficiency, congenital |
| WBC | 2q21.3 | rs35837297 | 2 | 136594439 | nonsynonymous SNV | *LCT* | 23103231 | AFR | 765 AfricanAmericans | Lactase deficiency, congenital |
| MON | 2q31.3 | rs1449263 | 2 | 182319301 | intergenic | *AC020595.1 (dist=28825), ITGA4 (dist=2633)* | 21738480 | EUR | 19,509 European ancestry individuals |  |
| MON | 2q31.3 | rs12988934 | 2 | 182323665 | intronic | *ITGA4* | 21738478 | ASN | 8,794 Japanese ancestry individuals |  |
| EOS | 2q34 | rs12619285 | 2 | 213824045 | ncRNA_intronic | *AC093865.1* | 19198610 | EUR | 9,392 Icelanders |  |
| MPV | 2q37.3 | rs4305276 | 2 | 241495013 | intronic | *ANKMY1* | 22139419 | EUR | 18,600 European ancestry individuals |  |
| PLT | 3p25.2 | rs7616006 | 3 | 12267648 | intergenic | *SYN2 (dist=34759), PPARG (dist=61218)* | 22139419 | EUR | 48,666 individuals of European ancestry | Obesity, severe; Obesity, resistance to; Insulin resistance, severe, digenic; Lipodystrophy, familial partial, type 3; Carotid intimal medial thickness 1; Diabetes, type 2 |
| PLT | 3p24.3 | rs7641175 | 3 | 18311412 | intronic | *TBC1D5* | 22139419 | EUR | 48,666 individuals of European ancestry |  |
| MCH | 3p24.2 | rs9310736 | 3 | 24350811 | intronic | *THRB* | 20139978/23222517 | EUR | Up to 14,362 Japanese ancestry individuals/62,553 European ancestry individuals, 9,308 South Asian ancestry individuals | Thyroid hormone resistance; Thyroid hormone resistance, autosomal recessive; Thyroid hormone resistance, selective pituitary |
| MCV | 3p24.2 | rs9310736 | 3 | 24350811 | intronic | *THRB* | 20139978/23222517 | EUR | 14,364 Japanese ancestry individuals/62,553 European ancestry individuals, 9,308 South Asian ancestry individuals | Thyroid hormone resistance; Thyroid hormone resistance, autosomal recessive; Thyroid hormone resistance, selective pituitary |
| MPV | 3p14.3 | rs1354034 | 3 | 56849749 | intronic | *ARHGEF3* | 22139419 | EUR | 18,600 European ancestry individuals |  |
| PLT | 3p14.3 | rs1354034 | 3 | 56849749 | intronic | *ARHGEF3* | 22139419/23263863/23263863/23263863 | EUR | 48,666 individuals of European ancestry/7,943 African American children, 6,234 European ancestry children/7,943 African American children, 6,234 European ancestry children/7,943 African American children, 6,234 European ancestry children |  |
| MPV | 3p14.3 | rs12485738 | 3 | 56865776 | intronic | *ARHGEF3* | 19110211/19820697 | EUR | 1,606 individuals/4,627 European individuals |  |
| PLT | 3q21.1 | rs3792366 | 3 | 122839876 | intronic | *PDIA5* | 22139419 | EUR | 48,666 individuals of European ancestry |  |
| MPV | 3q21.2 | rs10512627 | 3 | 124340222 | intronic | *KALRN* | 22139419 | EUR | 18,600 European ancestry individuals | Coronary heart disease, susceptibility to |
| EOS | 3q21.3 | rs4857855 | 3 | 128260550 | intergenic | *RP11-475N22.5 (dist=3076), C3orf27 (dist=30293)* | 19198610 | EUR | 9,392 Icelanders |  |
| MON | 3q21.3 | rs9880192 | 3 | 128297569 | intergenic | *C3orf27 (dist=2640), RPN1 (dist=41248)* | 21738480 | EUR | 19,509 European ancestry individuals |  |
| EOS | 3q21.3 | rs4328821 | 3 | 128316435 | intergenic | *C3orf27 (dist=21506), RPN1 (dist=22382)* | 21738478 | ASN | 8,794 Japanese ancestry individuals |  |
| BAS | 3q21.3 | rs4328821 | 3 | 128316435 | intergenic | *C3orf27 (dist=21506), RPN1 (dist=22382)* | 21738480/21738478 | EUR | 19,509 European ancestry individuals/8,794 Japanese ancestry individuals |  |
| MCV | 3q22 | rs6776003 | 3 | 141266493 | intronic | *RASA2* | 23222517 | EUR | 62,553 European ancestry individuals, 9,308 South Asian ancestry individuals |  |
| MCV | 3q23 | rs13061823 | 3 | 142120786 | intronic | *XRN1* | 23222517 | EUR | 62,553 European ancestry individuals, 9,308 South Asian ancestry individuals |  |
| PLT | 3q27.1 | rs6141 | 3 | 184090266 | UTR3 | *THPO* | 20139978 | ASN | 14,806 Japanese ancestry individuals | Thrombocythemia |
| MCV | 3q29 | rs9859260 | 3 | 195800547 | intronic | *TFRC* | 19862010 | EUR | 24,167 European ancestry individuals |  |
| MCH | 3q29 | rs11915082 | 3 | 195809139 | upstream | *TFRC* | 19862010 | EUR | 24,167 European ancestry individuals |  |
| MCH | 3q29 | rs11717368 | 3 | 195834357 | intergenic | *TFRC (dist=25297), AC139666.1 (dist=35150)* | 23222517 | EUR | 62,553 European ancestry individuals, 9,308 South Asian ancestry individuals |  |
| MCH | 3q29 | rs4916483 | 3 | 195907653 | intergenic | *AC139666.1 (dist=19892), ZDHHC19 (dist=16667)* | 20139978 | ASN | Up to 14,362 Japanese ancestry individuals |  |
| MCV | 3q29 | rs4916483 | 3 | 195907653 | intergenic | *AC139666.1 (dist=19892), ZDHHC19 (dist=16667)* | 20139978 | ASN | 14,364 Japanese ancestry individuals |  |
| MPV | 4p16.1 | rs11734132 | 4 | 6891519 | intergenic | *KIAA0232 (dist=5628), TBC1D14 (dist=19450)* | 22139419 | EUR | 18,600 European ancestry individuals |  |
| MCH | 4q12 | rs218237 | 4 | 55394172 | intergenic | *RP11-545H22.1 (dist=81282), AC006552.1 (dist=75206)* | 20139978 | ASN | Up to 14,362 Japanese ancestry individuals |  |
| MCV | 4q12 | rs218237 | 4 | 55394172 | intergenic | *RP11-545H22.1 (dist=81282), AC006552.1 (dist=75206)* | 20139978 | ASN | 14,364 Japanese ancestry individuals |  |
| RBC | 4q12 | rs218237 | 4 | 55394172 | intergenic | *RP11-545H22.1 (dist=81282), AC006552.1 (dist=75206)* | 20139978 | ASN | 14,392 Japanese ancestry individuals |  |
| RBC | 4q12 | rs218238 | 4 | 55395024 | intergenic | *RP11-545H22.1 (dist=82134), AC006552.1 (dist=74354)* | 23222517 | EUR | 62,553 European ancestry individuals, 9,308 South Asian ancestry individuals |  |
| MCV | 4q12 | rs172629 | 4 | 55407762 | intergenic | *RP11-545H22.1 (dist=94872), AC006552.1 (dist=61616)* | 19862010/20139978 | EUR | 24,167 European ancestry individuals/14,364 Japanese ancestry individuals |  |
| WBC | 4q13.3 | rs1371799 | 4 | 74977837 | intergenic | *CXCL2 (dist=12827), MTHFD2L (dist=2054)* | 21738479 | AFR | 16,388 African American individuals |  |
| PLT | 4q22.1 | rs7694379 | 4 | 88186509 | ncRNA_intronic | *RP11-529H2.1* | 22139419 | EUR | 48,666 individuals of European ancestry |  |
| MCV | 4q27 | rs13152701 | 4 | 122751061 | intronic | *BBS7* | 23222517 | EUR | 62,553 European ancestry individuals, 9,308 South Asian ancestry individuals | Bardet-Biedl syndrome |
| MCHC | 5p15.33 | rs4580814 | 5 | 1113244 | intergenic | *SLC12A7 (dist=1092), CTD-3080P12.3 (dist=60012)* | 20139978 | ASN | 14,377 Japanese ancestry individuals |  |
| RBC | 5p15.33 | rs2736100 | 5 | 1286516 | intronic | *TERT* | 20139978 | ASN | 14,392 Japanese ancestry individuals | Bone marrow failure, telomere-related; Dyskeratosis congenita, autosomal recessive ; Dyskeratosis congenita, autosomal dominant ; Coronary artery disease; Pulmonary fibrosis, telomere-related; Leukemia, acute myeloid;Melanoma, cutaneous malignant |
| MPV | 5q13.3 | rs2227831 | 5 | 76023494 | intronic | *F2R* | 22139419 | EUR | 18,600 European ancestry individuals |  |
| PLT | 5q13.3 | rs17568628 | 5 | 76046939 | intergenic | *F2R (dist=15333), F2RL1 (dist=67819)* | 22139419 | EUR | 48,666 individuals of European ancestry |  |
| MPV | 5q14.3 | rs4521516 | 5 | 88099951 | intronic | *MEF2C* | 22139419 | EUR | 18,600 European ancestry individuals | Mental retardation, stereotypic movements, epilepsy, and/or cerebral malformations; Chromosome 5q14.3 deletion syndrome |
| PLT | 5q14.3 | rs700585 | 5 | 88152117 | intronic | *MEF2C* | 22139419 | EUR | 48,666 individuals of European ancestry | Mental retardation, stereotypic movements, epilepsy, and/or cerebral malformations; Chromosome 5q14.3 deletion syndrome |
| PLT | 5q31.1 | rs2070729 | 5 | 131819921 | intronic | *IRF1* | 22139419 | EUR | 48,666 individuals of European ancestry | Myelodysplastic syndrome, preleukemic; Myelogenous leukemia, acute; Gastric cancer, somatic; Nonsmall cell lung cancer, somatic |
| EOS | 5q31.1 | rs4143832 | 5 | 131862977 | intergenic | *IRF1 (dist=36487), IL5 (dist=14159)* | 19198610 | EUR | 9,392 Icelanders |  |
| MPV | 5q33.3 | rs10076782 | 5 | 158604963 | intronic | *RNF145* | 22139419 | EUR | 18,600 European ancestry individuals |  |
| MCH | 6p23 | rs6914805 | 6 | 16281187 | intronic | *GMPR* | 23222517 | EUR | 62,553 European ancestry individuals, 9,308 South Asian ancestry individuals |  |
| PLT | 6p22.2 | rs12526480 | 6 | 25533534 | intronic | *LRRC16A* | 22423221/22423221 | AFR | 16,388 African American individuals/16388 African American |  |
| PLT | 6p22.2 | rs441460 | 6 | 25548288 | intronic | *LRRC16A* | 22139419 | EUR | 48,666 individuals of European ancestry |  |
| MCH | 6p22.2 | rs17342717 | 6 | 25821770 | intronic | *SLC17A1* | 20927387 | EUR | 3,012 European ancestry individuals |  |
| MCH | 6p22.2 | rs1408272 | 6 | 25842951 | intronic | *SLC17A3* | 19853236/19862010/23222517 | EUR | 2,538 Australian individuals, 3,477 Dutch individuals/24,167 European ancestry individuals/62,553 European ancestry individuals, 9,308 South Asian ancestry individuals | Uric acid concentration, serum, QTL; Gout susceptibility |
| HB | 6p22.2 | rs1800562 | 6 | 26093141 | nonsynonymous SNV | *HFE* | 19862010 | EUR | 24,167 European ancestry individuals | Hemochromatosis; Microvascular complications of diabetes ; Porphyria variegata, susceptibility to; Porphyria cutanea tarda, susceptibility to; Alzheimer disease, susceptibility to; Transferrin serum level QTL |
| HCT | 6p22.2 | rs1800562 | 6 | 26093141 | nonsynonymous SNV | *HFE* | 19862010 | EUR | 24,167 European ancestry individuals | Hemochromatosis; Microvascular complications of diabetes ; Porphyria variegata, susceptibility to; Porphyria cutanea tarda, susceptibility to; Alzheimer disease, susceptibility to; Transferrin serum level QTL |
| MCV | 6p22.2 | rs1800562 | 6 | 26093141 | nonsynonymous SNV | *HFE* | 19862010/19820697 | EUR | 24,167 European ancestry individuals/4,627 European individuals | Hemochromatosis; Microvascular complications of diabetes ; Porphyria variegata, susceptibility to; Porphyria cutanea tarda, susceptibility to; Alzheimer disease, susceptibility to; Transferrin serum level QTL |
| MCH | 6p22.2 | rs1800562 | 6 | 26093141 | nonsynonymous SNV | *HFE* | 20927387/20927387/23263863 | EUR | 3,012 European ancestry individuals/3,012 European ancestry individuals/7,943 African American children, 6,234 European ancestry children | Hemochromatosis; Microvascular complications of diabetes ; Porphyria variegata, susceptibility to; Porphyria cutanea tarda, susceptibility to; Alzheimer disease, susceptibility to; Transferrin serum level QTL |
| HB | 6p22.2 | rs198846 | 6 | 26107463 | downstream | *HIST1H1T* | 19820698 | EUR | 6,316 Europeans, 9,685 Indian Asians |  |
| MCH | 6p22.1 | rs13219787 | 6 | 27861670 | upstream | *HIST1H2BO* | 23222517 | EUR | 62,553 European ancestry individuals, 9,308 South Asian ancestry individuals |  |
| HB | 6p22.1 | rs2097775 | 6 | 30354303 | intergenic | *UBQLN1P (dist=22246), MICC (dist=28189)* | 23222517 | EUR | 62,553 European ancestry individuals, 9,308 South Asian ancestry individuals |  |
| WBC | 6p21.33 | rs2517510 | 6 | 31030122 | intergenic | *HCG22 (dist=2469), C6orf15 (dist=48878)* | 21738480 | EUR | 19,509 European ancestry individuals |  |
| WBC | 6p21.33 | rs3094212 | 6 | 31085770 | intronic | *CDSN* | 20139978 | ASN | 14,677 Japanese ancestry individuals | Hypotrichosis simplex of scalp; Peeling skin syndrome |
| MON | 6p21.33 | rs3095254 | 6 | 31221668 | intergenic | *HCG27 (dist=49923), HLA-C (dist=14858)* | 21738478 | ASN | 8,794 Japanese ancestry individuals | Psoriasis susceptibility ; HIV-1 viremia, susceptibility to |
| LYM | 6p21.33 | rs2524079 | 6 | 31242174 | intergenic | *HLA-C (dist=2292), USP8P (dist=1175)* | 21738480 | EUR | 19,509 European ancestry individuals | Psoriasis susceptibility ; HIV-1 viremia, susceptibility to |
| PLT | 6p21.33 | rs3819299 | 6 | 31322367 | intronic | *HLA-B* | 22139419 | EUR | 48,666 individuals of European ancestry | Spondyloarthropathy, susceptibility to; Abacavir hypersensitivity, susceptibility to; Synovitis, chronic, susceptibility to; Drug-induced liver injury due to flucloxacillin; Toxic epidermal necrolysis, susceptibility to; Stevens-Johnson syndrome, susceptibility to |
| EOS | 6p21.33 | rs2516399 | 6 | 31481299 | intergenic | *MICB (dist=2398), PPIAP9 (dist=5958)* | 21738478 | ASN | 8,794 Japanese ancestry individuals |  |
| WBC | 6p21.33 | rs389884 | 6 | 31940897 | intronic | *STK19* | 23263863 | EUR | 7,943 African American children, 6,234 European ancestry children |  |
| RBC | 6p21.32 | rs9272219 | 6 | 32602269 | intronic | *HLA-DQA1* | 23222517 | EUR | 62,553 European ancestry individuals, 9,308 South Asian ancestry individuals | Celiac disease, susceptibility to |
| PLT | 6p21.32 | rs399604 | 6 | 32975014 | intronic | *HLA-DOA* | 22139419 | EUR | 48,666 individuals of European ancestry |  |
| PLT | 6p21.31 | rs210134 | 6 | 33540209 | downstream | *BAK1* | 22139419/23263863/22423221 | EUR | 48,666 individuals of European ancestry/7,943 African American children, 6,234 European ancestry children/16,388 African American individuals |  |
| PLT | 6p21.31 | rs513349 | 6 | 33541719 | intronic | *BAK1* | 23103231/23263863 | AFR | 768 AfricanAmericans/7,943 African American children, 6,234 European ancestry children |  |
| PLT | 6p21.31 | rs5745568 | 6 | 33548394 | upstream | *BAK1* | 20139978 | ASN | 14,806 Japanese ancestry individuals |  |
| PLT | 6p21.31 | rs210135 | 6 | 33572915 | UTR3 | *BAK1* | 19820697 | EUR | 4,627 European individuals |  |
| MCH | 6p21.1 | rs3218097 | 6 | 41905275 | intronic | *CCND3* | 20139978 | ASN | Up to 14,362 Japanese ancestry individuals |  |
| MCV | 6p21.1 | rs3218097 | 6 | 41905275 | intronic | *CCND3* | 20139978 | ASN | 14,364 Japanese ancestry individuals |  |
| RBC | 6p21.1 | rs3218097 | 6 | 41905275 | intronic | *CCND3* | 20139978 | ASN | 14,392 Japanese ancestry individuals |  |
| MCV | 6p21.1 | rs9349204 | 6 | 41914378 | intronic | *CCND3* | 23222517 | EUR | 62,553 European ancestry individuals, 9,308 South Asian ancestry individuals |  |
| MCH | 6p21.1 | rs9349205 | 6 | 41925159 | intronic | *CCND3* | 19862010 | EUR | 24,167 European ancestry individuals |  |
| MCV | 6p21.1 | rs9349205 | 6 | 41925159 | intronic | *CCND3* | 19862010 | EUR | 24,167 European ancestry individuals |  |
| MCV | 6p21.1 | rs11970772 | 6 | 41925290 | intronic | *CCND3* | 19820697 | EUR | 4,627 European individuals |  |
| HB | 6p21.1 | rs9369427 | 6 | 43811430 | intergenic | *VEGFA (dist=57206), RP11-344J7.2 (dist=8064)* | 23222517 | EUR | 62,553 European ancestry individuals, 9,308 South Asian ancestry individuals |  |
| MCH | 6q21 | rs9386791 | 6 | 109608497 | intergenic | *RP11-352J4.2 (dist=16280), RP11-425D10.1 (dist=1146)* | 23446634 | AFR | Up to 16,485 African American individuals |  |
| MCV | 6q21 | rs9374080 | 6 | 109616420 | ncRNA_intronic | *C6orf184* | 19862010 | EUR | 24,167 European ancestry individuals |  |
| MCH | 6q21 | rs1008084 | 6 | 109626965 | ncRNA_intronic | *C6orf184* | 23222517 | EUR | 62,553 European ancestry individuals, 9,308 South Asian ancestry individuals |  |
| MCH | 6q21 | rs11966072 | 6 | 109634828 | ncRNA_intronic | *C6orf184* | 20139978 | ASN | Up to 14,362 Japanese ancestry individuals |  |
| MCV | 6q21 | rs11966072 | 6 | 109634828 | ncRNA_intronic | *C6orf184* | 20139978 | ASN | 14,364 Japanese ancestry individuals |  |
| RBC | 6q21 | rs11966072 | 6 | 109634828 | ncRNA_intronic | *C6orf184* | 20139978 | ASN | 14,392 Japanese ancestry individuals |  |
| MCHC | 6q23.3 | rs7775698 | 6 | 135418635 | intronic | *HBS1L* | 20139978 | ASN | 14,377 Japanese ancestry individuals |  |
| PLT | 6q23.3 | rs7775698 | 6 | 135418635 | intronic | *HBS1L* | 20139978 | ASN | 14,806 Japanese ancestry individuals |  |
| MCH | 6q23.3 | rs7775698 | 6 | 135418635 | intronic | *HBS1L* | 19853236/20139978/20927387/23263863 | EUR | 2,538 Australian individuals, 3,477 Dutch individuals/Up to 14,362 Japanese ancestry individuals/3,012 European ancestry individuals/7,943 African American children, 6,234 European ancestry children |  |
| MCV | 6q23.3 | rs7775698 | 6 | 135418635 | intronic | *HBS1L* | 19853236/20139978/23263863 | EUR | 2,538 Australian individuals, 3,477 Dutch individuals/14,364 Japanese ancestry individuals/7,943 African American children, 6,234 European ancestry children |  |
| RBC | 6q23.3 | rs7775698 | 6 | 135418635 | intronic | *HBS1L* | 20139978/20927387 | EUR | 14,392 Japanese ancestry individuals/3,012 European ancestry individuals |  |
| MCH | 6q23.3 | rs7776054 | 6 | 135418916 | intronic | *HBS1L* | 19862010 | EUR | 24,167 European ancestry individuals |  |
| MCH | 6q23.3 | rs9399137 | 6 | 135419018 | intronic | *HBS1L* | 23263863 | EUR | 7,943 African American children, 6,234 European ancestry children |  |
| MCV | 6q23.3 | rs9399137 | 6 | 135419018 | intronic | *HBS1L* | 23263863 | EUR | 7,943 African American children, 6,234 European ancestry children |  |
| PLT | 6q23.3 | rs9399137 | 6 | 135419018 | intronic | *HBS1L* | 19853236/22139419 | EUR | 2,538 Australian individuals, 3,477 Dutch individuals/48,666 individuals of European ancestry |  |
| EOS | 6q23.3 | rs9373124 | 6 | 135423209 | intronic | *HBS1L* | 21738478 | ASN | 8,794 Japanese ancestry individuals |  |
| WBC | 6q23.3 | rs4895441 | 6 | 135426573 | intergenic | *HBS1L (dist=2378), MYB (dist=75880)* | 20139978 | ASN | 14,677 Japanese ancestry individuals |  |
| MCV | 6q23.3 | rs4895441 | 6 | 135426573 | intergenic | *HBS1L (dist=2378), MYB (dist=75880)* | 19862010 | EUR | 24,167 European ancestry individuals |  |
| MCV | 6q23.3 | rs9389269 | 6 | 135427159 | intergenic | *HBS1L (dist=2964), MYB (dist=75294)* | 23222517 | EUR | 62,553 European ancestry individuals, 9,308 South Asian ancestry individuals |  |
| MCV | 6q23.3 | rs9402686 | 6 | 135427817 | intergenic | *HBS1L (dist=3622), MYB (dist=74636)* | 19820697 | EUR | 4,627 European individuals |  |
| PLT | 6q23.3 | rs9494145 | 6 | 135432552 | intergenic | *HBS1L (dist=8357), MYB (dist=69901)* | 22423221 | AFR | 16,388 African American individuals |  |
| MCV | 6q23.3 | rs9494145 | 6 | 135432552 | intergenic | *HBS1L (dist=8357), MYB (dist=69901)* | 20927387 | EUR | 3,012 European ancestry individuals |  |
| HCT | 6q23.3 | rs9483788 | 6 | 135435501 | intergenic | *HBS1L (dist=11306), MYB (dist=66952)* | 19862010 | EUR | 24,167 European ancestry individuals |  |
| MCH | 6q23.3 | rs6569992 | 6 | 135452152 | intergenic | *HBS1L (dist=27957), MYB (dist=50301)* | 20927387 | EUR | 3,012 European ancestry individuals |  |
| MCV | 6q23.3 | rs6569992 | 6 | 135452152 | intergenic | *HBS1L (dist=27957), MYB (dist=50301)* | 20927387 | EUR | 3,012 European ancestry individuals |  |
| RBC | 6q23.3 | rs6569992 | 6 | 135452152 | intergenic | *HBS1L (dist=27957), MYB (dist=50301)* | 20927387 | EUR | 3,012 European ancestry individuals |  |
| MCH | 6q24.1 | rs632057 | 6 | 139834012 | intergenic | *RP11-12A2.3 (dist=39820), RP11-15H7.1 (dist=101563)* | 20139978 | ASN | Up to 14,362 Japanese ancestry individuals |  |
| MCV | 6q24.1 | rs632057 | 6 | 139834012 | intergenic | *RP11-12A2.3 (dist=39820), RP11-15H7.1 (dist=101563)* | 20139978 | ASN | 14,364 Japanese ancestry individuals |  |
| MCH | 6q24.1 | rs668459 | 6 | 139835689 | intergenic | *RP11-12A2.3 (dist=41497), RP11-15H7.1 (dist=99886)* | 23263863 | EUR | 7,943 African American children, 6,234 European ancestry children |  |
| MCV | 6q24.1 | rs668459 | 6 | 139835689 | intergenic | *RP11-12A2.3 (dist=41497), RP11-15H7.1 (dist=99886)* | 23263863 | EUR | 7,943 African American children, 6,234 European ancestry children |  |
| MCH | 6q24.1 | rs628751 | 6 | 139838419 | intergenic | *RP11-12A2.3 (dist=44227), RP11-15H7.1 (dist=97156)* | 19862010 | EUR | 24,167 European ancestry individuals |  |
| MCV | 6q24.1 | rs643381 | 6 | 139839423 | intergenic | *RP11-12A2.3 (dist=45231), RP11-15H7.1 (dist=96152)* | 19862010 | EUR | 24,167 European ancestry individuals |  |
| MCV | 6q24.1 | rs590856 | 6 | 139844429 | intergenic | *RP11-12A2.3 (dist=50237), RP11-15H7.1 (dist=91146)* | 23222517 | EUR | 62,553 European ancestry individuals, 9,308 South Asian ancestry individuals |  |
| MCH | 6q26 | rs736661 | 6 | 164482836 | intergenic | *RP1-230L10.1 (dist=301963), RP1-155D22.1 (dist=46816)* | 23222517 | EUR | 62,553 European ancestry individuals, 9,308 South Asian ancestry individuals |  |
| MCV | 7p12.2 | rs12718597 | 7 | 50428428 | intronic | *IKZF1* | 19862010 | EUR | 24,167 European ancestry individuals | Leukemia, acute lymphoblastic |
| MCV | 7p12.2 | rs12718598 | 7 | 50428445 | intronic | *IKZF1* | 23222517 | EUR | 62,553 European ancestry individuals, 9,308 South Asian ancestry individuals | Leukemia, acute lymphoblastic |
| PLT | 7q21.11 | rs13236689 | 7 | 80236014 | intronic | *CD36* | 22423221/22423221 | AFR | 16,388 African American individuals/16388 African American | Macrothrombocytopenia; Platelet glycoprotein IV deficiency; Malaria, cerebral, susceptibility to; Malaria, cerebral, reduced risk of; Coronary heart disease, susceptibility to |
| WBC | 7q21.2 | rs445 | 7 | 92408370 | intronic | *CDK6* | 20139978 | ASN | 14,677 Japanese ancestry individuals |  |
| NEU | 7q21.2 | rs445 | 7 | 92408370 | intronic | *CDK6* | 21738478 | ASN | 8,794 Japanese ancestry individuals |  |
| MCV | 7q22.1 | rs7786877 | 7 | 100214015 | intergenic | *MOSPD3 (dist=1008), TFR2 (dist=4028)* | 19862010 | EUR | 24,167 European ancestry individuals |  |
| RBC | 7q22.1 | rs7385804 | 7 | 100235970 | intronic | *TFR2* | 19820697 | EUR | 4,627 European individuals | Hemochromatosis |
| HCT | 7q22.1 | rs7385804 | 7 | 100235970 | intronic | *TFR2* | 19862010 | EUR | 24,167 European ancestry individuals | Hemochromatosis |
| RBC | 7q22.1 | rs2075672 | 7 | 100240296 | intronic | *TFR2* | 23222517 | EUR | 62,553 European ancestry individuals, 9,308 South Asian ancestry individuals | Hemochromatosis |
| PLT | 7q22.3 | rs342275 | 7 | 106359216 | ncRNA_intronic | *CTB-111H14.1* | 22139419 | EUR | 48,666 individuals of European ancestry |  |
| PLT | 7q22.3 | rs342293 | 7 | 106372219 | ncRNA_intronic | *CTB-111H14.1* | 22423221 | AFR | 16,388 African American individuals |  |
| MPV | 7q22.3 | rs342293 | 7 | 106372219 | ncRNA_intronic | *CTB-111H14.1* | 22139419/19221038/19820697 | EUR | 18,600 European ancestry individuals/1,221 individuals/4,627 European individuals |  |
| MPV | 7q22.3 | rs342296 | 7 | 106372903 | upstream | *CTB-111H14.1* | 22423221 | AFR | 16,388 African American individuals |  |
| PLT | 7q31.32 | rs4731120 | 7 | 123411223 | intergenic | *WASL (dist=22102), HYALP1 (dist=42970)* | 22139419 | EUR | 48,666 individuals of European ancestry |  |
| HB | 7q36.1 | rs10480300 | 7 | 151406005 | intronic | *PRKAG2* | 23222517 | EUR | 62,553 European ancestry individuals, 9,308 South Asian ancestry individuals | Wolff-Parkinson-White syndrome; Cardiomyopathy, familial hypertrophic; Glycogen storage disease of heart, lethal congenital |
| HB | 7q36.1 | rs10224002 | 7 | 151415041 | intronic | *PRKAG2* | 19862010 | EUR | 24,167 European ancestry individuals | Wolff-Parkinson-White syndrome; Cardiomyopathy, familial hypertrophic; Glycogen storage disease of heart, lethal congenital |
| HCT | 7q36.1 | rs10224002 | 7 | 151415041 | intronic | *PRKAG2* | 19862010 | EUR | 24,167 European ancestry individuals | Wolff-Parkinson-White syndrome; Cardiomyopathy, familial hypertrophic; Glycogen storage disease of heart, lethal congenital |
| MCV | 8p21.3 | rs7843479 | 8 | 21820813 | intronic | *XPO7* | 20139978 | ASN | 14,364 Japanese ancestry individuals |  |
| MCHC | 8p11 | rs4737009 | 8 | 41630405 | intronic | *ANK1* | 23222517 | EUR | 62,553 European ancestry individuals, 9,308 South Asian ancestry individuals | Spherocytosis, type 1 |
| MCHC | 8p11 | rs6987853 | 8 | 42457450 | intergenic | *C8orf40 (dist=49299), RP11-359P18.1 (dist=840595)* | 23222517 | EUR | 62,553 European ancestry individuals, 9,308 South Asian ancestry individuals |  |
| PLT | 8q23.1 | rs6993770 | 8 | 106581528 | intronic | *ZFPM2* | 22139419 | EUR | 48,666 individuals of European ancestry | Tetralogy of Fallot; Diaphragmatic hernia |
| MON | 8q24.21 | rs10956483 | 8 | 130572110 | intronic | *CCDC26* | 21738478 | ASN | 8,794 Japanese ancestry individuals |  |
| MON | 8q24.21 | rs10098310 | 8 | 130613614 | intronic | *CCDC26* | 21738480 | EUR | 19,509 European ancestry individuals |  |
| PLT | 8q24.3 | rs6995402 | 8 | 145005561 | intronic | *PLEC* | 22139419 | EUR | 48,666 individuals of European ancestry | Muscular dystrophy with epidermolysis bullosa simplex; Epidermolysis bullosa simplex, Ogna type; Epidermolysis bullosa simplex with pyloric atresia; Muscular dystrophy, limb-girdle, type 2Q |
| MPV | 9p24.3 | rs10813766 | 9 | 331490 | intronic | *DOCK8* | 22139419 | EUR | 18,600 European ancestry individuals | Mental retardation, autosomal dominant; Hyper-IgE recurrent infection syndrome, autosomal recessive |
| PLT | 9p24.1 | rs409801 | 9 | 4744743 | intergenic | *AK3 (dist=2700), RP11-307I14.2 (dist=36732)* | 22139419 | EUR | 48,666 individuals of European ancestry |  |
| PLT | 9p24.1 | rs385893 | 9 | 4763176 | intergenic | *AK3 (dist=21133), RP11-307I14.2 (dist=18299)* | 20139978/20139978/19820697 | EUR | 14,806 Japanese ancestry individuals/14,806 Japanese ancestry individuals/4,627 European individuals |  |
| PLT | 9p24.1 | rs13300663 | 9 | 4814948 | intronic | *RCL1* | 22139419 | EUR | 48,666 individuals of European ancestry |  |
| MCH | 9p24.1 | rs2236496 | 9 | 4844265 | intronic | *RCL1* | 20139978/23222517 | EUR | Up to 14,362 Japanese ancestry individuals/62,553 European ancestry individuals, 9,308 South Asian ancestry individuals |  |
| MCV | 9p24.1 | rs2236496 | 9 | 4844265 | intronic | *RCL1* | 20139978/23222517 | EUR | 14,364 Japanese ancestry individuals/62,553 European ancestry individuals, 9,308 South Asian ancestry individuals |  |
| MCH | 9p24.1 | rs10758658 | 9 | 4856877 | intronic | *RCL1* | 19862010 | EUR | 24,167 European ancestry individuals |  |
| MCV | 9p24.1 | rs10758658 | 9 | 4856877 | intronic | *RCL1* | 19862010 | EUR | 24,167 European ancestry individuals |  |
| PLT | 9p21.3 | rs3731211 | 9 | 21986847 | intronic | *CDKN2A* | 22139419 | EUR | 48,666 individuals of European ancestry | Melanoma, cutaneous malignant; Melanoma and neural system tumor syndrome; Pancreatic cancer/melanoma syndrome; Orolaryngeal cancer, multiple |
| MON | 9q31.3 | rs10980800 | 9 | 113915905 | ncRNA_intronic | *RP11-202G18.1* | 21738480 | EUR | 19,509 European ancestry individuals |  |
| MCHC | 9q34.2 | rs8176746 | 9 | 136131322 | ncRNA_exonic | *ABO* | 20139978 | ASN | 14,377 Japanese ancestry individuals | Blood group, ABO system |
| RBC | 9q34.2 | rs579459 | 9 | 136154168 | intergenic | *ABO (dist=3551), LCN1L2 (dist=30461)* | 23222517 | EUR | 62,553 European ancestry individuals, 9,308 South Asian ancestry individuals | Blood group, ABO system |
| RBC | 9q34.2 | rs495828 | 9 | 136154867 | intergenic | *ABO (dist=4250), LCN1L2 (dist=29762)* | 20139978 | ASN | 14,392 Japanese ancestry individuals | Blood group, ABO system |
| PLT | 9q34.2 | rs11789898 | 9 | 136925663 | intronic | *BRD3* | 22139419 | EUR | 48,666 individuals of European ancestry |  |
| MCH | 10q11.21 | rs2279434 | 10 | 45955064 | intronic | *MARCH8* | 20139978 | ASN | Up to 14,362 Japanese ancestry individuals |  |
| MCV | 10q11.21 | rs2279434 | 10 | 45955064 | intronic | *MARCH8* | 20139978 | ASN | 14,364 Japanese ancestry individuals |  |
| MCV | 10q11.21 | rs901683 | 10 | 45966422 | intronic | *MARCH8* | 23222517 | EUR | 62,553 European ancestry individuals, 9,308 South Asian ancestry individuals |  |
| MCV | 10q11.21 | rs11239550 | 10 | 46024729 | intronic | *MARCH8* | 19862010 | EUR | 24,167 European ancestry individuals |  |
| MCH | 10q11.23 | rs7085433 | 10 | 51593354 | intronic | *TIMM23* | 20139978 | ASN | Up to 14,362 Japanese ancestry individuals |  |
| MCV | 10q11.23 | rs7085433 | 10 | 51593354 | intronic | *TIMM23* | 20139978 | ASN | 14,364 Japanese ancestry individuals |  |
| PLT | 10q21.3 | rs10761731 | 10 | 65027610 | intronic | *JMJD1C* | 22139419 | EUR | 48,666 individuals of European ancestry |  |
| MPV | 10q21.3 | rs7075195 | 10 | 65050659 | intronic | *JMJD1C* | 22139419 | EUR | 18,600 European ancestry individuals |  |
| PLT | 10q21.3 | rs7896518 | 10 | 65104500 | intronic | *JMJD1C* | 22423221/22423221 | AFR | 16,388 African American individuals/16388 African American |  |
| MPV | 10q21.3 | rs2393967 | 10 | 65133156 | intronic | *JMJD1C* | 19820697 | EUR | 4,627 European individuals |  |
| HB | 10q22.1 | rs16926246 | 10 | 71093392 | intronic | *HK1* | 19862010 | EUR | 24,167 European ancestry individuals | Hemolytic anemia due to hexokinase deficiency; Neuropathy, hereditary motor and sensory, Russe type |
| HCT | 10q22.1 | rs16926246 | 10 | 71093392 | intronic | *HK1* | 19862010 | EUR | 24,167 European ancestry individuals | Hemolytic anemia due to hexokinase deficiency; Neuropathy, hereditary motor and sensory, Russe type |
| HB | 10q21.3 | rs10159477 | 10 | 71099888 | intronic | *HK1* | 23222517 | EUR | 62,553 European ancestry individuals, 9,308 South Asian ancestry individuals | Hemolytic anemia due to hexokinase deficiency; Neuropathy, hereditary motor and sensory, Russe type |
| MCH | 10q24.2 | rs11190134 | 10 | 101282200 | intergenic | *RP11-441O15.3 (dist=86658), RP11-129J12.1 (dist=4638)* | 23222517 | EUR | 62,553 European ancestry individuals, 9,308 South Asian ancestry individuals |  |
| MPV | 11p15.5 | rs11602954 | 11 | 202856 | intronic | *BET1L* | 19820697 | EUR | 4,627 European individuals |  |
| PLT | 11p15.5 | rs505404 | 11 | 243268 | intronic | *PSMD13* | 22139419 | EUR | 48,666 individuals of European ancestry |  |
| MPV | 11p15.5 | rs17655730 | 11 | 270715 | intergenic | *PSMD13 (dist=17732), NLRP6 (dist=7855)* | 22139419 | EUR | 18,600 European ancestry individuals |  |
| MCHC | 11p15.4 | rs7120391 | 11 | 5230907 | intergenic | *AC104389.16 (dist=2369), HBB (dist=15787)* | 23696099 | AFR | 1904 Afican American individuals |  |
| HCT | 11p15.4 | rs334 | 11 | 5248231 | nonsynonymous SNV | *HBB* | 23103231 | AFR | 761 AfricanAmericans | Sickle cell anemia; Thalassemias, beta-; Erythremias, beta- ; Methemoglobinemias, beta- ; Heinz body anemias, beta-; Thalassemia-beta, dominant inclusion-body; Hereditary persistence of fetal hemoglobin; Delta-beta thalassemia; Malaria, resistance to |
| HCT | 11p15.4 | rs2213169 | 11 | 5303063 | intronic | *HBG2* | 23263863 | AFR | 7,943 African American children, 6,234 European ancestry children | Fetal hemoglobin quantitative trait locus ; Cyanosis, transient neonatal |
| MCHC | 11p15.4 | rs2213169 | 11 | 5303063 | intronic | *HBG2* | 23263863 | AFR | 7,943 African American children, 6,234 European ancestry children | Fetal hemoglobin quantitative trait locus ; Cyanosis, transient neonatal |
| fHB | 11p15.4 | rs4910742 | 11 | 5306509 | intronic | *HBG2* | 18245381 | EUR | 4,305Sardinians | Fetal hemoglobin quantitative trait locus ; Cyanosis, transient neonatal |
| fHB | 11p15.4 | rs5006884 | 11 | 5373251 | nonsynonymous SNV | *OR51B6* | 20018918 | AFR | 848 Black cases |  |
| HB | 11p15.4 | rs11042125 | 11 | 8938049 | intronic | *AKIP1* | 23222517 | EUR | 62,553 European ancestry individuals, 9,308 South Asian ancestry individuals |  |
| HCT | 11p15.4 | rs7936461 | 11 | 10040886 | intronic | *SBF2* | 23222517 | EUR | 62,553 European ancestry individuals, 9,308 South Asian ancestry individuals | Charcot-Marie-Tooth disease, type 4B2 |
| PLT | 11q12.2 | rs4246215 | 11 | 61564299 | UTR3 | *FEN1* | 22139419 | EUR | 48,666 individuals of European ancestry |  |
| PLT | 11q13.1 | rs477895 | 11 | 64048912 | intronic | *BAD* | 22423221/22423221 | AFR | 16,388 African American individuals/16388 African American |  |
| MCV | 11q13.2 | rs2302264 | 11 | 67207426 | intronic | *CORO1B* | 23222517 | EUR | 62,553 European ancestry individuals, 9,308 South Asian ancestry individuals |  |
| HB | 11q13.4 | rs7125949 | 11 | 73009084 | UTR3 | *P2RY6* | 23222517 | EUR | 62,553 European ancestry individuals, 9,308 South Asian ancestry individuals |  |
| BAS | 11q14.3 | rs11018874 | 11 | 89875437 | intronic | *NAALAD2* | 21738478 | ASN | 8,794 Japanese ancestry individuals |  |
| PLT | 11q23.3 | rs4938642 | 11 | 119099906 | intronic | *CBL* | 22139419 | EUR | 48,666 individuals of European ancestry | Noonan syndrome-like disorder with or without juvenile myelomonocytic leukemia |
| HCT | 12p13.33 | rs7312105 | 12 | 2523355 | intronic | *CACNA1C* | 23222517 | EUR | 62,553 European ancestry individuals, 9,308 South Asian ancestry individuals | Timothy syndrome; Brugada syndrome |
| MCH | 12p13.32 | rs10849023 | 12 | 4332478 | intergenic | *RP11-320N7.1 (dist=113097), RP11-264F23.1 (dist=101060)* | 23222517 | EUR | 62,553 European ancestry individuals, 9,308 South Asian ancestry individuals |  |
| RBC | 12p13.32 | rs11611647 | 12 | 4333919 | intergenic | *RP11-320N7.1 (dist=114538), RP11-264F23.1 (dist=99619)* | 20139978 | ASN | 14,392 Japanese ancestry individuals |  |
| MPV | 12p13.31 | rs1558324 | 12 | 6289219 | intergenic | *VWF (), CD9 (dist=19662)* | 22139419 | EUR | 18,600 European ancestry individuals |  |
| PLT | 12p13.31 | rs7342306 | 12 | 6291093 | intergenic | *KCNA1 (dist=1269013), CD9 (dist=17788)* | 22139419 | EUR | 48,666 individuals of European ancestry |  |
| MPV | 12p11.22 | rs2015599 | 12 | 29435480 | intronic | *RP11-996F15.2* | 22139419 | EUR | 18,600 European ancestry individuals |  |
| MPV | 12q13.13 | rs10876550 | 12 | 54712308 | intronic | *RP11-968A15.8* | 22139419 | EUR | 18,600 European ancestry individuals |  |
| PLT | 12q13.13 | rs4326844 | 12 | 54736470 | intronic | *RP11-968A15.8* | 23263863 | EUR | 7,943 African American children, 6,234 European ancestry children |  |
| PLT | 12q13.3 | rs941207 | 12 | 57023284 | intronic | *BAZ2A* | 22139419 | EUR | 48,666 individuals of European ancestry |  |
| MPV | 12q13.3 | rs2950390 | 12 | 57055291 | intergenic | *ATP5B (dist=15493), PTGES3 (dist=1835)* | 22139419 | EUR | 18,600 European ancestry individuals |  |
| WBC | 12q15 | rs12313946 | 12 | 68961584 | intergenic | *RP11-254B13.1 (dist=13922), RAP1B (dist=43104)* | 20139978 | ASN | 14,677 Japanese ancestry individuals |  |
| RBC | 12q22 | rs11104870 | 12 | 88829294 | intergenic | *Y_RNA (dist=4966), KITLG (dist=56591)* | 23222517 | EUR | 62,553 European ancestry individuals, 9,308 South Asian ancestry individuals |  |
| EOS |  | rs3184504 | 12 | 111884608 | synonymous SNV | *SH2B3* | 19198610 | EUR | 9,392 Icelanders | Myelofibrosis, somatic; Thrombocythemia, somatic; Erythrocytosis, somatic |
| PLT | 12q24.12 | rs3184504 | 12 | 111884608 | nonsynonymous SNV | *SH2B3* | 22139419 | EUR | 48,666 individuals of European ancestry | Myelofibrosis, somatic; Thrombocythemia, somatic; Erythrocytosis, somatic |
| HB | 12q24.12 | rs3184504 | 12 | 111884608 | nonsynonymous SNV | *SH2B3* | 23222517 | EUR | 62,553 European ancestry individuals, 9,308 South Asian ancestry individuals | Myelofibrosis, somatic; Thrombocythemia, somatic; Erythrocytosis, somatic |
| PLT | 12q24.12 | rs739496 | 12 | 111887659 | UTR3 | *SH2B3* | 20139978 | ASN | 14,806 Japanese ancestry individuals | Myelofibrosis, somatic; Thrombocythemia, somatic; Erythrocytosis, somatic |
| PLT | 12q24.12 | rs11065987 | 12 | 112072424 | intergenic | *BRAP (dist=8372), ATXN2 (dist=34944)* | 19820697 | EUR | 4,627 European individuals |  |
| HB | 12q24.12 | rs11065987 | 12 | 112072424 | intergenic | *BRAP (dist=8372), ATXN2 (dist=34944)* | 19862010 | EUR | 24,167 European ancestry individuals |  |
| HCT | 12q24.12 | rs11065987 | 12 | 112072424 | intergenic | *BRAP (dist=8372), ATXN2 (dist=34944)* | 19862010 | EUR | 24,167 European ancestry individuals |  |
| PLT | 12q24.12 | rs6490294 | 12 | 112190438 | intronic | *ACAD10* | 22423221 | AFR | 16,388 African American individuals |  |
| MCHC | 12q24.12 | rs671 | 12 | 112241766 | nonsynonymous SNV | *ALDH2* | 20139978 | ASN | 14,377 Japanese ancestry individuals | Alcohol sensitivity, acute; Hangover, susceptibility to; Sublingual nitroglycerin, susceptibility to poor response to; Esophageal cancer, alcohol-related, susceptibility to |
| PLT | 12q24.13 | rs11066301 | 12 | 112871372 | intronic | *PTPN11* | 19820697 | EUR | 4,627 European individuals | Noonan syndrome; LEOPARD syndrome ; Leukemia, juvenile myelomonocytic; Metachondromatosis |
| PLT | 12q24.13 | rs17824620 | 12 | 113100994 | intronic | *RPH3A* | 22139419 | EUR | 48,666 individuals of European ancestry |  |
| PLT | 12q24.31 | rs7961894 | 12 | 122365583 | intronic | *WDR66* | 22139419 | EUR | 48,666 individuals of European ancestry |  |
| MPV | 12q24.31 | rs7961894 | 12 | 122365583 | intronic | *WDR66* | 22139419/19110211/19820697 | EUR | 18,600 European ancestry individuals/1,606 individuals/4,627 European individuals |  |
| PLT | 13q32.1 | rs4148441 | 13 | 95898207 | intronic | *ABCC4* | 22139419 | EUR | 48,666 individuals of European ancestry |  |
| MPV | 13q34 | rs7317038 | 13 | 114012898 | intronic | *GRTP1-AS1* | 22139419 | EUR | 18,600 European ancestry individuals |  |
| MON | 14q12 | rs10147992 | 14 | 25503799 | intronic | *STXBP6* | 21738478 | ASN | 8,794 Japanese ancestry individuals |  |
| MCV | 14q23.3 | rs4466998 | 14 | 65475540 | intronic | *FNTB* | 19862010 | EUR | 24,167 European ancestry individuals |  |
| PLT | 14q24.1 | rs8022206 | 14 | 68520906 | intronic | *RAD51B* | 22139419 | EUR | 48,666 individuals of European ancestry |  |
| MCV | 14q24.2 | rs11627546 | 14 | 70365924 | intronic | *SMOC1* | 23222517 | EUR | 62,553 European ancestry individuals, 9,308 South Asian ancestry individuals | Microphthalmia with limb anomalies |
| PLT | 14q32.12 | rs8006385 | 14 | 93501026 | intronic | *ITPK1* | 22139419 | EUR | 48,666 individuals of European ancestry |  |
| PLT | 14q32.2 | rs7149242 | 14 | 101159416 | intergenic | *RP11-566J3.2 (dist=13194), DLK1 (dist=32626)* | 22139419 | EUR | 48,666 individuals of European ancestry |  |
| PLT | 14q32.31 | rs11628318 | 14 | 103040087 | intergenic | *RP11-796G6.1 (dist=895370), RP11-661D19.1 (dist=88490)* | 22139419 | EUR | 48,666 individuals of European ancestry |  |
| PLT | 14q32.32 | rs2297067 | 14 | 103566785 | nonsynonymous SNV | *EXOC3L4* | 22139419 | EUR | 48,666 individuals of European ancestry |  |
| MPV | 14q32.32 | rs944002 | 14 | 103572815 | intronic | *EXOC3L4* | 22139419 | EUR | 18,600 European ancestry individuals |  |
| MCH | 14q32.32 | rs17616316 | 14 | 103822762 | intergenic | *EIF5 (dist=11399), HMGB3P26 (dist=7814)* | 23222517 | EUR | 62,553 European ancestry individuals, 9,308 South Asian ancestry individuals |  |
| MPV | 14q32.33 | rs3000073 | 14 | 105729792 | intronic | *BRF1* | 22139419 | EUR | 18,600 European ancestry individuals |  |
| HB | 15q21 | rs1532085 | 15 | 58683366 | intronic | *ALDH1A2* | 23222517 | EUR | 62,553 European ancestry individuals, 9,308 South Asian ancestry individuals |  |
| PLT | 15q22.2 | rs3809566 | 15 | 63333724 | intergenic | *TLN2 (dist=196894), TPM1 (dist=1107)* | 22139419 | EUR | 48,666 individuals of European ancestry | Cardiomyopathy, familial hypertrophic; Cardiomyopathy, dilated, 1Y; Left ventricular noncompaction |
| MPV | 15q22.2 | rs11071720 | 15 | 63341996 | intronic | *TPM1* | 19820697 | EUR | 4,627 European individuals | Cardiomyopathy, familial hypertrophic; Cardiomyopathy, dilated, 1Y; Left ventricular noncompaction |
| PLT | 15q22.31 | rs1719271 | 15 | 65183801 | intronic | *CTD-2017F17.1* | 22139419 | EUR | 48,666 individuals of European ancestry |  |
| MCH | 15q22.31 | rs6494537 | 15 | 66051345 | intronic | *DENND4A* | 20139978 | ASN | Up to 14,362 Japanese ancestry individuals |  |
| MCV | 15q22.31 | rs2572207 | 15 | 66070693 | intronic | *DENND4A* | 23222517 | EUR | 62,553 European ancestry individuals, 9,308 South Asian ancestry individuals |  |
| MCV | 15q24.2 | rs8028632 | 15 | 75321262 | intronic | *PPCDC* | 23222517 | EUR | 62,553 European ancestry individuals, 9,308 South Asian ancestry individuals |  |
| HB | 15q24.2 | rs11072566 | 15 | 76293971 | intronic | *NRG4* | 23222517 | EUR | 62,553 European ancestry individuals, 9,308 South Asian ancestry individuals |  |
| MCHC | 15q25.1 | rs2867932 | 15 | 78591037 | intronic | *WDR61* | 23222517 | EUR | 62,553 European ancestry individuals, 9,308 South Asian ancestry individuals |  |
| MCH | 16p13.3 | rs11248850 | 16 | 163598 | intronic | *NPRL3* | 23222517 | EUR | 62,553 European ancestry individuals, 9,308 South Asian ancestry individuals |  |
| HB | 16p13.3 | rs7203560 | 16 | 184390 | intronic | *NPRL3* | 23263863 | AFR | 7,943 African American children, 6,234 European ancestry children |  |
| MCH | 16p13.3 | rs7203560 | 16 | 184390 | intronic | *NPRL3* | 23263863 | AFR | 7,943 African American children, 6,234 European ancestry children |  |
| MCHC | 16p13.3 | rs7203560 | 16 | 184390 | intronic | *NPRL3* | 23263863 | AFR | 7,943 African American children, 6,234 European ancestry children |  |
| MCV | 16p13.3 | rs7203560 | 16 | 184390 | intronic | *NPRL3* | 23263863 | AFR | 7,943 African American children, 6,234 European ancestry children |  |
| RBC | 16p13.3 | rs7203560 | 16 | 184390 | intronic | *NPRL3* | 23263863 | AFR | 7,943 African American children, 6,234 European ancestry children |  |
| MCH | 16p13.3 | rs2858942 | 16 | 225653 | intergenic | *HBA2 (dist=1944), HBA1 (dist=1026)* | 20139978 | ASN | Up to 14,362 Japanese ancestry individuals | Thalassemias, alpha-; Methemoglobinemias, alpha-; Erythremias, alpha- ; Heinz body anemias, alpha-; Hemoglobin H disease, nondeletional |
| HB | 16p13.3 | rs11863726 | 16 | 230578 | synonymous SNV | *HBQ1* | 23103231 | AFR | 764 AfricanAmericans |  |
| MCH | 16p13.3 | rs1211375 | 16 | 240280 | intronic | *LUC7L* | 23263863 | AFR | 7,943 African American children, 6,234 European ancestry children |  |
| MCV | 16p13.3 | rs1211375 | 16 | 240280 | intronic | *LUC7L* | 23263863 | EUR | 7,943 African American children, 6,234 European ancestry children |  |
| RBC | 16p13.3 | rs1211375 | 16 | 240280 | intronic | *LUC7L* | 23263863 | EUR | 7,943 African American children, 6,234 European ancestry children |  |
| MCH | 16p13.3 | rs13339636 | 16 | 298588 | intronic | *ITFG3* | 23446634 | AFR | Up to 16,485 African American individuals |  |
| MCV | 16p13.3 | rs7189020 | 16 | 304803 | intronic | *ITFG3* | 19862010 | EUR | 24,167 European ancestry individuals |  |
| MCHC | 16p13.3 | rs1122794 | 16 | 309155 | intronic | *ITFG3* | 23446634 | AFR | Up to 16,485 African American individuals |  |
| MCH | 16p13.3 | rs1122794 | 16 | 309155 | intronic | *ITFG3* | 19862010 | EUR | 24,167 European ancestry individuals |  |
| HB | 16p13.3 | rs13335497 | 16 | 310005 | synonymous SNV | *ITFG3* | 23103231 | AFR | 763 African Americans |  |
| HB | 16p13.3 | rs13335629 | 16 | 310380 | intronic | *ITFG3* | 23446634 | AFR | Up to 16,485 African American individuals |  |
| MCHC | 16p13.3 | rs13335629 | 16 | 310380 | intronic | *ITFG3* | 23446634 | AFR | Up to 16,485 African American individuals |  |
| MCV | 16p13.3 | rs13335629 | 16 | 310380 | intronic | *ITFG3* | 23446634 | AFR | Up to 16,485 African American individuals |  |
| RBC | 16p13.3 | rs13335629 | 16 | 310380 | intronic | *ITFG3* | 23446634 | AFR | Up to 16,485 African American individuals |  |
| HB | 16p13.3 | rs9924561 | 16 | 314780 | intronic | *ITFG3* | 23103231 | AFR | 762 AfricanAmericans |  |
| MCH | 16p13.3 | rs9924561 | 16 | 314780 | intronic | *ITFG3* | 23696099 | AFR | 1904 Afican American individuals |  |
| MCHC | 16p13.3 | rs9924561 | 16 | 314780 | intronic | *ITFG3* | 23696099 | AFR | 1904 Afican American individuals |  |
| MCV | 16p13.3 | rs9924561 | 16 | 314780 | intronic | *ITFG3* | 23696099 | AFR | 1904 Afican American individuals |  |
| MCHC | 16p13.3 | rs2266928 | 16 | 580124 | intronic | *SOLH* | 23263863 | EUR | 7,943 African American children, 6,234 European ancestry children |  |
| RBC | 16q22.1 | rs2271294 | 16 | 67902326 | intronic | *NUTF2* | 23222517 | EUR | 62,553 European ancestry individuals, 9,308 South Asian ancestry individuals |  |
| MCHC | 16q24.3 | rs10445033 | 16 | 88840462 | intronic | *PIEZO1* | 23222517 | EUR | 62,553 European ancestry individuals, 9,308 South Asian ancestry individuals | Dehydrated hereditary stomatocytosis with or without pseudohyperkalemia and/or perinatal edema |
| MCHC | 16q24.3 | rs837763 | 16 | 88853729 | intergenic | *PIEZO1 (dist=2109), GALNS (dist=26413)* | 20139978 | ASN | 14,377 Japanese ancestry individuals | Dehydrated hereditary stomatocytosis with or without pseudohyperkalemia and/or perinatal edema |
| PLT | 17p13.2 | rs6065 | 17 | 4836381 | nonsynonymous SNV | *GP1BA* | 22139419/20139978 | EUR | 48,666 individuals of European ancestry/14,806 Japanese ancestry individuals | Bernard-Soulier syndrome, type A1 (recessive); Nonarteritic anterior ischemic optic neuropathy, susceptibility to; Bernard-Soulier syndrome, type A2 (dominant); von Willebrand disease, platelet-type |
| PLT | 17p11.2 | rs397969 | 17 | 19804247 | intergenic | *ULK2 (dist=32998), AKAP10 (dist=3541)* | 22139419 | EUR | 48,666 individuals of European ancestry | Cardiac conduction defect, susceptibility to |
| MCH | 17p11.2 | rs888424 | 17 | 19985427 | intronic | *SPECC1* | 23222517 | EUR | 62,553 European ancestry individuals, 9,308 South Asian ancestry individuals |  |
| MCH | 17p11.2 | rs2070265 | 17 | 27075423 | synonymous SNV | *TRAF4* | 23222517 | EUR | 62,553 European ancestry individuals, 9,308 South Asian ancestry individuals |  |
| MPV | 17q11.2 | rs11653144 | 17 | 27675226 | intergenic | *RP11-22N12.1 (dist=7386), GIT1 (dist=225261)* | 22423221 | AFR | 16,388 African American individuals |  |
| MPV | 17q11.2 | rs2138852 | 17 | 27703349 | intronic | *RP11-296K13.4* | 19110211/19820697 | EUR | 1,606 individuals/4,627 European individuals |  |
| MPV | 17q11.2 | rs8076739 | 17 | 27714587 | intronic | *RP11-296K13.4* | 22139419 | EUR | 18,600 European ancestry individuals |  |
| PLT | 17q11.2 | rs559972 | 17 | 27814496 | intronic | *TAOK1* | 22139419 | EUR | 48,666 individuals of European ancestry |  |
| PLT | 17q12 | rs10512472 | 17 | 33884804 | nonsynonymous SNV | *SLFN14* | 22139419 | EUR | 48,666 individuals of European ancestry |  |
| MPV | 17q12 | rs16971217 | 17 | 33944055 | intronic | *AP2B1* | 22139419 | EUR | 18,600 European ancestry individuals |  |
| RBC | 17q12 | rs8182252 | 17 | 37727950 | intergenic | *CDK12 (dist=6789), NEUROD2 (dist=32071)* | 23222517 | EUR | 62,553 European ancestry individuals, 9,308 South Asian ancestry individuals |  |
| WBC | 17q21.1 | rs17609240 | 17 | 38110689 | intergenic | *ORMDL3 (dist=26835), GSDMA (dist=8536)* | 19820697 | EUR | 4,627 European individuals |  |
| WBC | 17q21.1 | rs3859192 | 17 | 38128648 | intronic | *GSDMA* | 22037903 | EUR | 62 American Indian or Alaska Native ancestry individuals, 158 Asian ancestry individuals, 3,272 African American ancestry individuals, 114 other ancestry individuals, 23,244 European ancestry individuals, 996 unknown ancestry individuals |  |
| WBC | 17q21.1 | rs4065321 | 17 | 38143548 | intronic | *PSMD3* | 20139978 | ASN | 14,677 Japanese ancestry individuals |  |
| NEU | 17q21.1 | rs4794822 | 17 | 38156712 | intergenic | *PSMD3 (dist=2499), CSF3 (dist=14902)* | 21738478 | ASN | 8,794 Japanese ancestry individuals |  |
| WBC | 17q21.1 | rs4794822 | 17 | 38156712 | intergenic | *PSMD3 (dist=2499), CSF3 (dist=14902)* | 21738480 | EUR | 19,509 European ancestry individuals |  |
| WBC | 17q21.1 | rs8078723 | 17 | 38166879 | intergenic | *PSMD3 (dist=12666), CSF3 (dist=4735)* | 22788528 | ASN | 8,722Korean |  |
| NEU | 17q21.1 | rs8078723 | 17 | 38166879 | intergenic | *PSMD3 (dist=12666), CSF3 (dist=4735)* | 21738480 | EUR | 19,509 European ancestry individuals |  |
| MCHC | 17q21.31 | rs2269906 | 17 | 42294337 | intronic | *UBTF* | 23222517 | EUR | 62,553 European ancestry individuals, 9,308 South Asian ancestry individuals |  |
| PLT | 17q21.31 | rs708382 | 17 | 42442344 | intergenic | *FAM171A2 (dist=1101), C17orf104 (dist=291428)* | 22139419 | EUR | 48,666 individuals of European ancestry |  |
| RBC | 17q21.31 | rs12150672 | 17 | 43826637 | intronic | *CRHR1* | 23222517 | EUR | 62,553 European ancestry individuals, 9,308 South Asian ancestry individuals |  |
| HB | 17q25 | rs4969184 | 17 | 76393413 | intronic | *PGS1* | 23222517 | EUR | 62,553 European ancestry individuals, 9,308 South Asian ancestry individuals |  |
| PLT | 18q11.2 | rs11082304 | 18 | 20720973 | intronic | *CABLES1* | 22139419 | EUR | 48,666 individuals of European ancestry |  |
| MCH | 18q21 | rs4890633 | 18 | 43833278 | intronic | *C18orf25* | 23222517 | EUR | 62,553 European ancestry individuals, 9,308 South Asian ancestry individuals |  |
| MPV | 18q22.2 | rs893001 | 18 | 67516845 | intronic | *CD226* | 19820697 | EUR | 4,627 European individuals |  |
| MPV | 18q22.2 | rs12969657 | 18 | 67536496 | intronic | *CD226* | 22139419 | EUR | 18,600 European ancestry individuals |  |
| HB | 19p13.3 | rs2159213 | 19 | 2136102 | intronic | *AP3D1* | 23222517 | EUR | 62,553 European ancestry individuals, 9,308 South Asian ancestry individuals |  |
| MCV | 19p13.3 | rs732716 | 19 | 4366219 | intronic | *SH3GL1* | 23222517 | EUR | 62,553 European ancestry individuals, 9,308 South Asian ancestry individuals | Leukemia, acute myeloid |
| MCV | 19p13.2 | rs7255045 | 19 | 12932269 | intronic | *HOOK2* | 19862010 | EUR | 24,167 European ancestry individuals |  |
| MCH | 19p13.2 | rs11085824 | 19 | 13001547 | intergenic | *KLF1 (6310), GCDH (426)* | 19862010 | EUR | 24,167 European ancestry individuals | Glutaricaciduria, type I |
| MCH | 19p13.2 | rs741702 | 19 | 13024250 | intronic | *SYCE2* | 23222517 | EUR | 62,553 European ancestry individuals, 9,308 South Asian ancestry individuals |  |
| PLT | 19p13.12 | rs8109288 | 19 | 16185559 | intronic | *TPM4* | 22139419/21153663/22423221 | EUR | 48,666 individuals of European ancestry/23,439 Caucasians/16,388 African American individuals |  |
| MPV | 19p13.12 | rs8109288 | 19 | 16185559 | intronic | *TPM4* | 22139419/22423221 | EUR | 18,600 European ancestry individuals/16,388 African American individuals |  |
| LYM | 19p13.11 | rs10411936 | 19 | 16548375 | intronic | *EPS15L1* | 21738480 | EUR | 19,509 European ancestry individuals |  |
| MCV | 19q13.11 | rs3892630 | 19 | 33181484 | intergenic | *RGS9BP (dist=12278), NUDT19 (dist=1382)* | 23222517 | EUR | 62,553 European ancestry individuals, 9,308 South Asian ancestry individuals |  |
| PLT | 19q13.32 | rs17356664 | 19 | 45740771 | intergenic | *EXOC3L2 (dist=5633), CTC-315K20.1 (dist=95185)* | 22139419 | EUR | 48,666 individuals of European ancestry |  |
| MCV | 19q13.43 | rs7258661 | 19 | 56691601 | intronic | *GALP* | 21153663 | EUR | 23,439 Caucasians |  |
| MPV | 20p13 | rs6136489 | 20 | 1923734 | intergenic | *SIRPA (dist=3191), RP4-684O24.5 (dist=4158)* | 19820697 | EUR | 4,627 European individuals |  |
| MPV | 20p13 | rs13042885 | 20 | 1924707 | intergenic | *SIRPA (dist=4164), RP4-684O24.5 (dist=3185)* | 22139419 | EUR | 18,600 European ancestry individuals |  |
| HB | 20q13.2 | rs6013509 | 20 | 51318351 | intergenic | *RP4-715N11.2 (dist=10883), RP11-80K6.2 (dist=156526)* | 19862010 | EUR | 24,167 European ancestry individuals |  |
| MCV | 20q13.31 | rs737092 | 20 | 55990405 | intergenic | *RBM38 (dist=6016), HMGB1L1 (dist=73043)* | 23222517 | EUR | 62,553 European ancestry individuals, 9,308 South Asian ancestry individuals |  |
| MCV | 20q13.31 | rs6092477 | 20 | 55991695 | intergenic | *RBM38 (dist=7306), HMGB1L1 (dist=71753)* | 20139978 | ASN | 14,364 Japanese ancestry individuals |  |
| MPV | 20q13.32 | rs4812048 | 20 | 57587771 | intergenic | *CTSZ (dist=5469), TUBB1 (dist=6538)* | 22139419 | EUR | 18,600 European ancestry individuals |  |
| PLT | 20q13.32 | rs151361 | 20 | 57614002 | intronic | *SLMO2* | 22423221/22423221 | AFR | 16,388 African American individuals/16388 African American |  |
| HCT | 21q22.11 | rs2032314 | 21 | 35354523 | intergenic | *AP000569.8 (dist=4531), MRPS6 (dist=91300)* | 23222517 | EUR | 62,553 European ancestry individuals, 9,308 South Asian ancestry individuals |  |
| WBC | 21q22.12 | rs16993221 | 21 | 36849419 | intronic | *RUNX1* | 22788528 | ASN | 8,722Korean | Leukemia, acute myeloid; Platelet disorder, familial, with associated myeloid malignancy |
| BAS | 21q22.2 | rs7275212 | 21 | 39852551 | intronic | *ERG* | 21738478 | ASN | 8,794 Japanese ancestry individuals |  |
| PLT | 22q11.21 | rs1034566 | 22 | 19984277 | intronic | *ARVCF* | 22139419 | EUR | 48,666 individuals of European ancestry |  |
| MCV | 22q11.21 | rs5754217 | 22 | 21939675 | intronic | *TOP3B, UBE2L3* | 23222517 | EUR | 62,553 European ancestry individuals, 9,308 South Asian ancestry individuals |  |
| MCV | 22q11.21 | rs4821112 | 22 | 21964761 | intronic | *UBE2L3* | 20139978 | ASN | 14,364 Japanese ancestry individuals |  |
| MCV | 22q12.3 | rs9609565 | 22 | 32867528 | intergenic | *BPIL2 (dist=7057), FBXO7 (dist=3135)* | 19820697 | EUR | 4,627 European individuals | Parkinson disease , autosomal recessive |
| MCH | 22q12.3 | rs5749446 | 22 | 32880585 | intronic | *FBXO7* | 23222517 | EUR | 62,553 European ancestry individuals, 9,308 South Asian ancestry individuals | Parkinson disease , autosomal recessive |
| MCHC | 22q12.3 | rs855791 | 22 | 37462936 | nonsynonymous SNV | *TMPRSS6* | 20139978 | ASN | 14,377 Japanese ancestry individuals | Iron-refractory iron deficiency anemia |
| HB | 22q12.3 | rs855791 | 22 | 37462936 | nonsynonymous SNV | *TMPRSS6* | 19820698/19862010/23263863 | EUR | 6,316 Europeans, 9,685 Indian Asians/24,167 European ancestry individuals/7,943 African American children, 6,234 European ancestry children | Iron-refractory iron deficiency anemia |
| MCH | 22q12.3 | rs855791 | 22 | 37462936 | nonsynonymous SNV | *TMPRSS6* | 20139978/20927387/23263863/23263863 | EUR | Up to 14,362 Japanese ancestry individuals/3,012 European ancestry individuals/7,943 African American children, 6,234 European ancestry children/7,943 African American children, 6,234 European ancestry children | Iron-refractory iron deficiency anemia |
| MCV | 22q12.3 | rs855791 | 22 | 37462936 | nonsynonymous SNV | *TMPRSS6* | 20139978/20927387/23263863/23263863 | EUR | 14,364 Japanese ancestry individuals/3,012 European ancestry individuals/7,943 African American children, 6,234 European ancestry children/7,943 African American children, 6,234 European ancestry children | Iron-refractory iron deficiency anemia |
| MCH | 22q12.3 | rs5756506 | 22 | 37467392 | intronic | *TMPRSS6* | 19820697 | EUR | 4,627 European individuals | Iron-refractory iron deficiency anemia |
| MCH | 22q12.3 | rs4820268 | 22 | 37469591 | synonymous SNV | *TMPRSS6* | 19853236 | EUR | 2,538 Australian individuals, 3,477 Dutch individuals | Iron-refractory iron deficiency anemia |
| MCV | 22q12.3 | rs4820268 | 22 | 37469591 | synonymous SNV | *TMPRSS6* | 19853236 | EUR | 2,538 Australian individuals, 3,477 Dutch individuals | Iron-refractory iron deficiency anemia |
| MCHC | 22q12.3 | rs4820268 | 22 | 37469591 | synonymous SNV | *TMPRSS6* | 20927387 | EUR | 3,012 European ancestry individuals | Iron-refractory iron deficiency anemia |
| HCT | 22q12.3 | rs2413450 | 22 | 37470224 | intronic | *TMPRSS6* | 19862010 | EUR | 24,167 European ancestry individuals | Iron-refractory iron deficiency anemia |
| MCH | 22q12.3 | rs2413450 | 22 | 37470224 | intronic | *TMPRSS6* | 19862010 | EUR | 24,167 European ancestry individuals | Iron-refractory iron deficiency anemia |
| MCV | 22q12.3 | rs2413450 | 22 | 37470224 | intronic | *TMPRSS6* | 19862010 | EUR | 24,167 European ancestry individuals | Iron-refractory iron deficiency anemia |
| MCH | 22q13.33 | rs470119 | 22 | 50966914 | intronic | *TYMP* | 20139978 | ASN | Up to 14,362 Japanese ancestry individuals | Mitochondrial DNA depletion syndrome 1 |
| MCV | 22q13.33 | rs140522 | 22 | 50971266 | upstream | *ODF3B* | 23222517 | EUR | 62,553 European ancestry individuals, 9,308 South Asian ancestry individuals |  |
| MCV | 22q13.33 | rs131794 | 22 | 50971752 | upstream | *ODF3B* | 19862010 | EUR | 24,167 European ancestry individuals |  |
| HB | 23q26 | rs1050828 | 23 | 153764217 | nonsynonymous SNV | *G6PD* | 21153663 | AFR | 7,112 African Americans | Hemolytic anemia due to G6PD deficiency; Favis; Resistance to malaria due to G6PD deficiency |
| HCT | 23q26 | rs1050828 | 23 | 153764217 | nonsynonymous SNV | *G6PD* | 21153663 | AFR | 7,112 African Americans | Hemolytic anemia due to G6PD deficiency; Favis; Resistance to malaria due to G6PD deficiency |
| MCH | 23q28 | rs1050828 | 23 | 153764217 | intronic | *G6PD* | 23696099 | AFR | 1904 Afican American individuals | Hemolytic anemia due to G6PD deficiency; Favis; Resistance to malaria due to G6PD deficiency |
| RBC | 23q28 | rs1050828 | 23 | 153764217 | intronic | *G6PD* | 23446634/23696099/21153663 | AFR | Up to 16,485 African American individuals/1904 Afican American individuals/ 7,112 African Americans | Hemolytic anemia due to G6PD deficiency; Favis; Resistance to malaria due to G6PD deficiency |
| MCV | 23q28 | rs1050828 | 23 | 153764217 | intronic | *G6PD* | 23696099/21153663 | AFR | 1904 Afican American individuals/ 7,112 African Americans | Hemolytic anemia due to G6PD deficiency; Favis; Resistance to malaria due to G6PD deficiency |
| HB | 23q28 | rs762516 | 23 | 153764663 | intronic | *G6PD* | 23446634 | AFR | Up to 16,485 African American individuals | Hemolytic anemia due to G6PD deficiency; Favis; Resistance to malaria due to G6PD deficiency |
| HCT | 23q28 | rs762516 | 23 | 153764663 | intronic | *G6PD* | 23446634 | AFR | Up to 16,485 African American individuals | Hemolytic anemia due to G6PD deficiency; Favis; Resistance to malaria due to G6PD deficiency |
| MCV | 23q28 | rs762516 | 23 | 153764663 | intronic | *G6PD* | 23446634 | AFR | Up to 16,485 African American individuals | Hemolytic anemia due to G6PD deficiency; Favis; Resistance to malaria due to G6PD deficiency |
| MCV | 23q28 | rs5987027 | 23 | 154014107 | intronic | *MPP1* | 23263863 | AFR | 7,943 African American children, 6,234 European ancestry children |  |
| RBC | 23q28 | rs5987027 | 23 | 154014107 | intronic | *MPP1* | 23263863 | AFR | 7,943 African American children, 6,234 European ancestry children |  |

**Table S2.**  Enrichment of genes related with Mendelian traits in blood GWAS lead SNPs.

We carried out Fisher’s exact test where we count genes related with any Mendelian trait or disease in a test panel consisting of nearest genes to 211 blood GWAS lead SNPs that are associated with platelets and red cells traits (PUBMED ID 22139419, 23222517). Similarly, we look for Mendelian trait-related genes in a background panel of 211 SNPs taken randomly from dbSNP version 138. We find significant enrichment in blood GWAS SNPs with fold change of 1.93 and *P* - value < 10^-3^.

| *Is the nearest gene related to a Mendelian trait?* | Blood GWAS SNP | Random SNP |
| --- | --- | --- |
| *Yes* | 54 | 28 |
| *No* | 157 | 183 |

**Table S3.** Website and PUBMED references for various databases, tools and experimental methods that are cited as examples in Figures 2 and 3 in the main text.

| **Database/Tools** | **Website / PUBMED ID** |
| --- | --- |
| 1000G | *http://www.1000genomes.org/* |
| Bioconductor | *http://www.bioconductor.org/* |
| BioGRID | *http://thebiogrid.org/* |
| Blueprint | *http://www.blueprint-epigenome.eu/* |
| Caviar | *http://genetics.cs.ucla.edu/caviar/* |
| ChromHMM | *22373907* |
| dbSNP | *http://www.ncbi.nlm.nih.gov/SNP/* |
| DMAP | *http://www.broadinstitute.org/dmap/home* |
| ENCODE | *https://www.encodeproject.org/* |
| Enlight | *http://enlight.usc.edu/* |
| ENSEMBL | *http://www.ensembl.org/* |
| FANTOM5 | *http://fantom.gsc.riken.jp/5/* |
| FuncSNP-seq | *http://bioinfo.cipf.es/funcsnp/download.html* |
| GATK | *https://www.broadinstitute.org/gatk/* |
| GCTA | *21167468* |
| GEMMA | *22706312* |
| GENCODE | *http://www.gencodegenes.org/* |
| Gene Ontology | *http://geneontology.org/* |
| GWAS3D | *23723249* |
| GWAVA | *https://www.sanger.ac.uk/sanger/StatGen_Gwava* |
| HaemAtlas | *http://haemgen.haem.cam.ac.uk/haematlas/* |
| Haploreg | *http://www.broadinstitute.org/mammals/haploreg/haploreg.php* |
| Haploview | *15297300* |
| HapMap | *http://hapmap.ncbi.nlm.nih.gov/* |
| KEGG | *http://www.genome.jp/kegg/* |
| Limix | *https://github.com/PMBio/limix* |
| Matrix eQTL | *22492648* |
| Meta-Tissue | *23785294* |
| PLINK | *17701901* |
| Reactome | *http://www.reactome.org/* |
| RegulomeDB | *http://www.regulomedb.org/* |
| ROADMAP epigenomics | *http://www.roadmapepigenomics.org/* |
| SNAP | *https://www.broadinstitute.org/mpg/snap/* |
| SNPTEST | *17572673* |
| UK10K | *http://www.uk10k.org/* |
| UniProt | *http://www.uniprot.org/* |
| Variant Effect Predictor | *http://www.ensembl.org/info/docs/tools/vep/index.html* |
| VariantAnnotation | 24681907 |
